# Declining temperature and increasing moisture sensitivity of shrub growth in the Low‐Arctic erect dwarf‐shrub tundra of western Greenland

**DOI:** 10.1002/ece3.9419

**Published:** 2022-11-06

**Authors:** Stef Weijers

**Affiliations:** ^1^ Department of Geography University of Bonn Bonn Germany

**Keywords:** branch mortality, branching, browning, greening, NDVI, shrub expansion, soil moisture, winter warming events

## Abstract

Evergreen dwarf shrubs respond swiftly to warming in the cool and dry High Arctic, but their response in the warmer Low Arctic, where they are expected to be outcompeted by taller species under future warming, remains to be clarified.

Here, 12,528 annual growth increments, covering 122 years (1893–2014), were measured of 764 branches from 25 individuals of the evergreen dwarf shrub *Cassiope tetragona* from a Low‐Arctic erect dwarf‐shrub tundra site in western Greenland. In addition, branch initiation and mortality frequency time series were developed. The influence of seasonal climate and correspondence with fluctuations in regional normalized difference vegetation index (NDVI), a satellite‐proxy for vegetation productivity, were studied.

Summer temperatures were the main driver of growth, while winter temperatures were the main non‐summer‐climate driver. During past and recent warm episodes, shrub growth diverged from summer temperatures. In recent decades, early summer precipitation has become the main growth‐limiting factor for some individuals, likely through micro‐topography‐determined soil moisture availability, and more than half of the shrubs studied became irresponsive to summer temperatures. There was correspondence between climatic drivers, *C. tetragona* growth and branch initiation frequency, and satellite‐observed vegetation productivity, suggesting the area's shrub‐dominated tundra vegetation is limited by similar climatic factors. Winter warming events were likely the predominant cause of branch mortality, while branching increased after years with poor growth and cooler‐than‐average summers.

These findings show that the erect dwarf‐shrub tundra in the Low Arctic has already and will likely become decreasingly temperature‐ and increasingly moisture‐limited and that winter warming supports shrub growth, but increased extreme winter warming event frequency may increase branch mortality and vegetation damage. Such counter‐acting mechanisms could offer an explanation for the vegetation stability observed over large parts of the Arctic.

## Graphical abstract

The erect dwarf‐shrub tundra in the Low Arctic has already and will likely become decreasingly temperature‐ and increasingly moisture‐limited. Winter warming supports shrub growth, but increased extreme winter warming event frequency increases branch mortality and vegetation damage. Such counter‐acting, balancing mechanisms could offer an explanation for the still widespread vegetation stability in large parts of the Arctic.

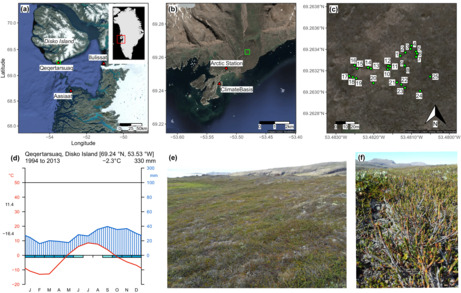

## 1 INTRODUCTION

Arctic and alpine areas are warming at faster rates than other areas (AMAP, 2021; IPCC, 2021), partly because of decreased albedo after advanced snowmelt (Pepin et al., 2015) and feedback mechanisms related to the decline in Arctic sea‐ice extent (Serreze & Barry, 2011). The Arctic tundra biome is divided into five bioclimatic subzones, which are defined by summer temperatures and dominant plant growth forms in each zone (Walker et al., 2016). The vertical tundra vegetation structure varies from a discontinuous layer of very short plants in the coldest zone to multi‐layered, complex canopies in the warmest zone. As a consequence of the rapid warming, the entirety of each of these vegetation zones is expected to shift northward and into higher elevations. Plant height has already increased over the past 30 years throughout the tundra biome, largely caused by species turnover due to immigration of taller species from warmer areas (Bjorkman et al., 2018). This is at least partially related to the increase in productivity and abundance of deciduous shrub species (Sturm et al., 2001). Such an increase in canopy height, because of increased abundance of tall shrub species, may lead to an increase in competition for light and a decrease in dwarf shrub abundance, particularly in the Low‐ and Sub‐Arctic tundra (Elmendorf et al., 2012). However, the potential of deciduous shrubs to increase with summer warming likely depends on soil moisture availability (Ackerman et al., 2017; Gamm et al., 2018; Myers‐Smith et al., 2015). Deciduous shrub growth has increased rapidly with recent warming in warmer wetter areas, while it has declined in dry tundra areas in recent decades, possibly in relation to diminishing sea‐ice extent (Buchwal et al., 2020). In the cooler and drier High Arctic, evergreen dwarf shrub growth has increased rapidly with recent warming (Weijers et al., 2017), and leaf size and plant height of evergreen dwarf shrubs increases to long‐term experimental warming (Hudson et al., 2011). However, it remains unknown whether evergreen dwarf shrubs, which are generally better adapted to drier conditions and are found on well‐drained, relatively dry soils (Rune, 2011), can respond with increased growth to the amplified warming in the already comparatively warm Low Arctic.

The satellite‐based normalized difference vegetation index (NDVI) is generally regarded as a proxy for biomass and productivity of terrestrial vegetation. NDVI analyses have revealed long‐term greening trends over large parts of the tundra biome in the northern hemisphere in recent decades (Goetz et al., 2005, 2011; Huang et al., 2017; Jia et al., 2003, 2009; Ju & Masek, 2016; Verbyla, 2008; Zhu et al., 2016). Nonetheless, a significant percentage of Arctic ecosystems appears to be stable and the mechanisms behind this stability are poorly understood (Callaghan et al., 2021). Interannual variation in NDVI in the Siberian Arctic (Blok et al., 2011; Forbes et al., 2010; Macias‐Fauria et al., 2012) and Arctic‐alpine northwest North America (Andreu‐Hayles et al., 2020; Tape et al., 2012; Weijers, Pape, et al., 2018) have been related to interannual variation in shrub growth and summer temperatures. This suggests a possible link between summer warming‐induced Arctic greening and shrub growth. Although many tundra areas have experienced greening over the past decades, other regions have experienced browning events in recent times (AMAP, 2017; Phoenix & Bjerke, 2016). Such browning events have been linked to the occurrence of extreme warm spells in winter, which result in snowmelt, often accompanied by rain‐on‐snow events (Peeters et al., 2019). These may result in the encasement of plants in ground ice or leave plants exposed to subsequent periods of severe frost without insulation by a sufficiently deep snow layer (Bjerke et al., 2017; Bokhorst et al., 2008). Extreme winter warming and rain‐on‐snow events, the frequency of which is likely to increase in the future (Hansen et al., 2014), can damage vegetation and lower its productivity in the subsequent summer (Bokhorst et al., 2009). Single events have been linked to branch mortality of several shrub species, including *Cassiope tetragona* (Bjerke et al., 2017; Milner et al., 2016). Still, shrub branch mortality and branch initiation frequency time series and possible links with climate and NDVI have not been studied thus far.

Here, the climate‐growth relationships in the evergreen dwarf shrub *Cassiope tetragona* are studied at a Low‐Arctic site in western Greenland. The species is found throughout the Arctic, but is most prevalent in the High Arctic (Walker et al., 2005). The following research questions were addressed:

- What are the main seasonal climate drivers of *C. tetragona* shrub growth at the site and individual shrub level and are these stable over time?

Summer temperatures were hypothesized to be the main driver of growth, as was observed at other Sub‐Arctic sites for this species (see Weijers et al., 2012; Weijers, Pape, et al., 2018). Winter temperatures were suspected to be potential co‐drivers of *C. tetragona* growth, as this was observed for *Betula nana* shrubs at an adjacent site (Hollesen et al., 2015).

ii Are there differences in individual shrub growth responses to climate and are these related to micro‐topographic positions?

As snow‐depth and related micro‐climatic factors such as soil temperature and moisture content are at least partly determined by micro‐topography, variation in growth responses between shrub individuals was hypothesized.

iii Is annual variation in branch initiation and mortality frequency related to variation in seasonal climate variables?

Branch initiation was hypothesized to be influenced by growing season climate, especially summer temperatures, similar to shrub growth. In contrast, branch mortality was expected to be related to winter climate, as in winter the evergreen leaves may be exposed and not survive the harsh Arctic conditions.

iv Are area‐wide, satellite‐observed vegetation productivity, *C. tetragona* growth, branch initiation and mortality frequencies, and seasonal climate factors inter‐related?

These factors were expected to be inter‐related, as *C. tetragona* was found to potentially be a proxy of annual above‐ground tundra vegetation productivity at the landscape level (Milner et al., 2018), and links between annual shoot length series of this species and NDVI have been observed at an Arctic‐alpine site in the Yukon, Canada (Weijers et al., 2012).

## 2 MATERIALS AND METHODS

### 2.1 Site description

**FIGURE 1:**
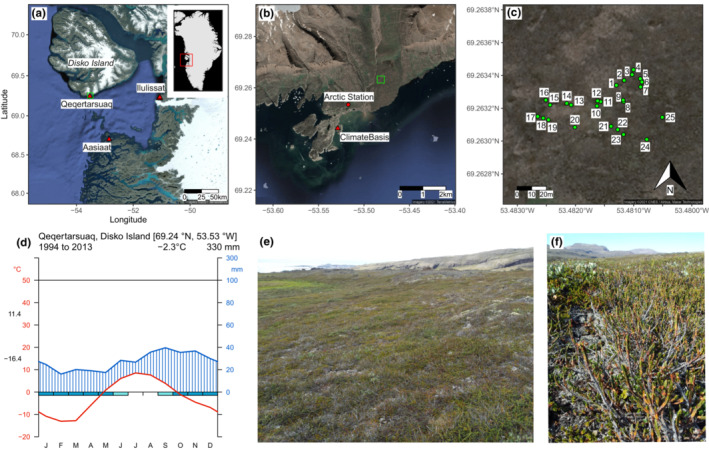
Location of the research site near Qeqertarsuaq on Disko Island (green square); weather station locations are indicated with red triangles (a). The inset map shows the location of the Disko Bay area (red square) within Greenland. Location of the research site (green square) northeast of Arctic Station (b). Local weather is recorded at ClimateBasis Disko, approx. 3 km southwest of the site. Individual shrub sampling spots (c). Climate diagram showing monthly mean air temperatures (red) and precipitation sums (blue) at Qeqertarsuaq for the period 1994–2013 (d). Values to the left of the temperature axis indicate the mean minimum temperature of the coldest month (February) and the mean maximum temperature of the warmest month (July). Photo of the research site taken from sample 4 in southwestern direction (e). Vegetation at sample spot 24 in north‐western direction dominated by *Cassiope tetragona* shrubs (f).

On 6 August 2014, 25 *Cassiope tetragona* shrubs were sampled at a mesic tundra site (69.26 °N, 53.48 °W, 45 m above sea level [m a.s.l.]; Figure 1a–c) located in the Blæsedalen valley about 2 km northeast of Arctic Station near Qeqertarsuaq on the southern tip of Disko Island, western Greenland. The area's soils consist of basaltic rock fragments, covered by a 5–10 cm organic horizon, while active layer depths can reach 1.5 m (Blok et al., 2016). The geology of the area is characterized by a low bench of gneiss bedrock, Tertiary breccia, and plateau lava (Hollesen et al., 2015). Shrub samples were collected in an area of approx. 60 m × 100 m. A minimum of 1–2 m distance were kept between sampling spots to avoid repeated sampling of the same shrub individual. It was attempted to sample complete (excl. roots) shrub individuals by following shrub branches until reaching the root‐shoot boundary.

The vegetation at the site can be described as erect dwarf‐shrub tundra, which covers 9.7 (13.7) percent, that is, 689 × 10^3^ km^2^, of the (non‐glaciated) Arctic (Walker et al., 2005). It is dominated by the deciduous shrub species *Betula nana*, *Salix glauca*, and *Vaccinium uliginosum* and the evergreen shrub species *Cassiope tetragona*, *Empetrum nigrum* ssp. *hermaphroditum*, and less frequently, *Rhododendron tomentosum*. The climate at the site is Low‐Arctic with a mean annual air temperature of −2.3°C measured over the period 1994–2013 at ClimateBasis Disko (Greenland Ecosystem Monitoring, 2020), 3 km southwest of the site, and an interpolated annual precipitation sum (CRU TS 4.04; Harris et al., 2020) of 330 mm over the same period (Figure 1d). February was the month with the lowest mean air temperature (−13.0°C), and March was the month with the lowest mean minimum air temperature (−16.4°C). July is the warmest month with a mean air temperature of 8.5°C and a mean maximum of 11.4°C. Mean monthly air temperatures remain below zero from November through April, hence most precipitation then falls as snow. Frosts are likely to occur as late as June and as early as September (Figure 1d). September is the wettest month with a mean precipitation sum of 39.6 mm and February is the driest (16.1 mm).

The site is located on a gentle south‐southeast‐facing slope (Figure 1e) at which *C. tetragona* is the dominant species (Figure 1f). Further west near Ilulissat on mainland Greenland, the species is only found on north‐facing slopes, while further south in the Disko Bay area, near Aasiaat, the species is absent at low elevations.

### 2.2 Species

**FIGURE A1:**
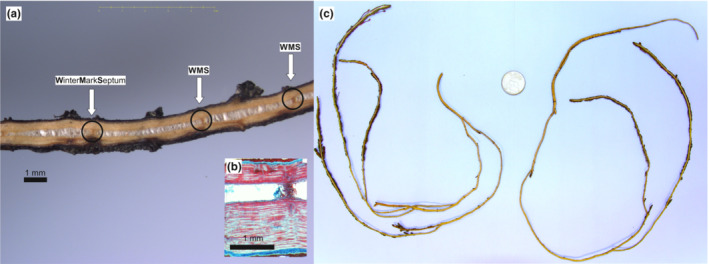
Lateral cross‐section of a *Cassiope tetragona* stem (a). The dark bands in the otherwise white pith are wintermarksepta (WMS). Lateral thin section of a *Cassiope tetragona* stem with a WMS stained with Astra Blue and Safranin (b). Red indicates lignified cell walls, while blue indicates parenchyma cells. Prepared sample (c). The sample (DI23) was divided into two subsamples. The coin (50 euro cent) has a diameter of 24.25 mm.

*Cassiope tetragona* (L.) D. Don., common name Arctic Bell Heather, is an evergreen dwarf shrub of the Ericaceae family. The species has a hemi‐prostrate growth form and reaches heights of up to 10–15 cm at the research site, but can grow up to 30 cm tall when supported by, for example, rocks. It is the dominant species in prostrate/hemi‐prostrate dwarf‐shrub tundra, but less so in the erect dwarf‐shrub tundra as found at the research site, and has a circumarctic distribution (Walker et al., 2005). It occurs in the northernmost polar desert at 83 °N in northern Greenland (Weijers et al., 2017), where it is the most common heath‐forming species (Rune, 2011). In western Greenland, it is found as far south as 63.35 °N, but towards the south only at alpine sites or on north‐facing slopes (Rune, 2011). The species' leaves are attached to its stems in opposite pairs, which alternate in 90° angles resulting in four rows of leaves (hence *tetragona*). Leaf lengths rapidly increase at the beginning of the growing season and decrease thereafter, resulting in a wave‐like pattern with each wave representing a single growing season (Warming, 1908). The discovery of so‐called wintermarksepta (WMS; Rozema et al., 2009), that is, dark bands coinciding with lows in leaf lengths visible in lateral cross‐sections of *C. tetragona* branches, has enabled the construction of up to 169‐year‐long growth chronologies (see for example Weijers et al., 2010). WMS consist of green and with increasing age, brown lignified meristem cells (see Figure A1).

### 2.3 Shrub growth chronologies

**FIGURE A2:**
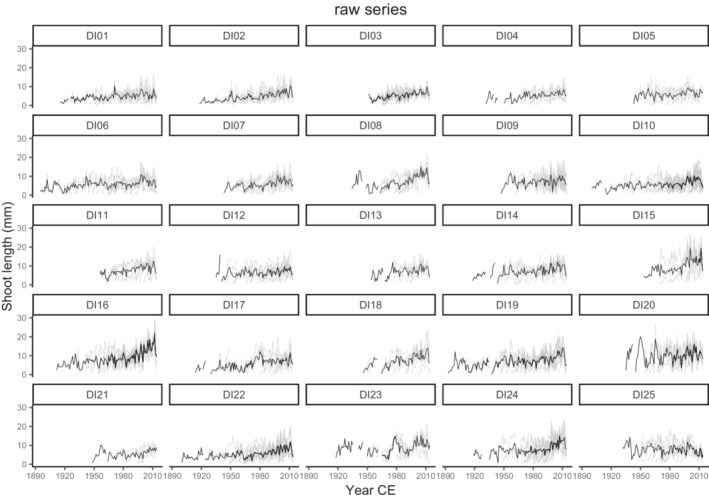
Individual raw branch series (gray lines) and mean annual shoot length growth (black lines) for the 25 *Cassiope tetragona* shrub samples from Disko Island, Greenland (DI01–DI25).

Thin layers were carefully removed with a scalpel from each branch, layer by layer, until the white pith and WMS therein became clearly visible within the branches (Figure A1). The shrub samples were kept intact as much as possible during this process, but sometimes samples were divided into smaller subsamples to simplify the layer removal. In such cases, the places where the subsamples were connected were marked. This was also done when a branch was accidentally cut into multiple parts. No branches had to be discarded due to irregularities. The sampling year, that is, the last year of growth, can be confidently identified as newly formed leaves are often brighter green, while older evergreen leaves after winter appear darker or more reddish green. Leaves of dead tips are brown and later gray. Leaves of dried *C. tetragona* samples retain their color for many years. Annual shoot length growth was measured as distances between WMS for multiple branches of each of the 25 sampled shrubs. This was done for a total of 764 branches, 10–65 per shrub. Shoot length growth series of individual branches were cross‐dated using the sampling year of shoots with complete and green tips, by plotting and visually comparing similarities in growth patterns between branch series of shrub individuals in MS Excel 2013, and the dates of branching. Older branch series, without any overlap with other series from the same shrub, were cross‐dated visually with dated branch series from other shrubs. This mainly concerned some series dating prior to the 1960s (cf. Figure A2; Weijers et al., 2010, 2017; Weijers, Wagner‐Cremer, et al., 2013).

**FIGURE 2:**
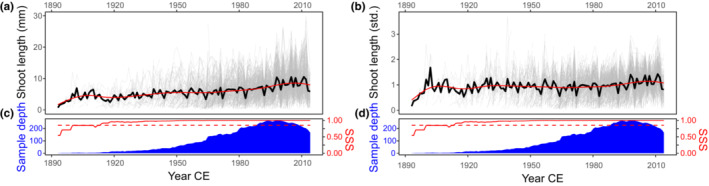
Raw (a) and standardized (b) shoot length growth series (gray lines) together with the site mean (black lines). The red lines through the site means are 30‐year cubic smoothing splines with a 50 percent frequency cut‐off, which indicate the longer‐term trends in the mean site chronologies. The individual branch series in (b) were standardized with a horizontal line through the mean. Sample depth (blue area) and subsample signal strength (SSS; Wigley et al., 1984; red lines) for the raw (c) and standardized (d) site chronology.

**FIGURE A3:**
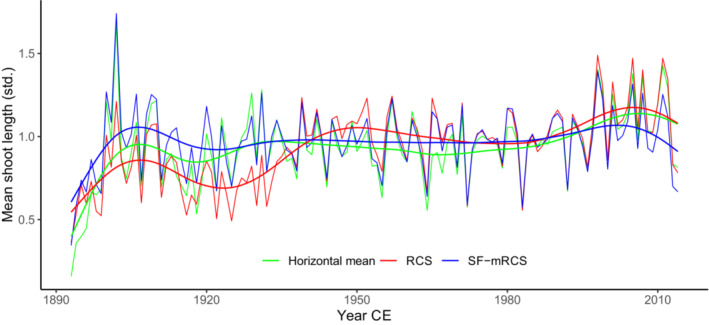
Trend comparison of the standardized *Cassiope tetragona* site chronologies constructed with three different standardization or detrending methods. Thick lines are 30‐year cubic smoothing splines with a 50 percent frequency cut‐off, which indicate the longer‐term trends in the mean site chronologies (thin lines).

Individual branch series of *C. tetragona* generally exhibit growth trends with increasing growth rates during the initial years of growth, approximately during the first 10 years in this study. Shoot length growth rates stabilize thereafter, before they start to decline when reaching ages of approx. 50 years (cf. Weijers, 2012). This may result in an overall positive trend in the mean site chronology (Figure 2) and mean shrub chronologies (Figure A2). Three different standardization or detrending methods were compared for this study (Table A1, Figure A3) to remove such growth trends: (1) standardization with a horizontal line through the mean (cf. Weijers et al., 2010), (2) Regional Curve Standardization (RCS; Briffa et al., 1992), and (3) signal‐free multiple RCS‐standardization (SF‐MRCS; Helama et al., 2017). With the first method, each individual branch series was divided by the related branch mean. In RCS‐detrending age‐related growth, trends are in principle removed, while lower‐frequency climatic trends are retained (Cook et al., 1995), with an age‐related growth curve. In SF‐MRCS detrending, multiple RCS curves are calculated for different groups to prevent potential bias resulting from e.g., differing growth rates of slow and fast‐growing individuals (Briffa & Melvin, 2011). Here, RCS curves were calculated for 11 groups (each with *n* > 50) of branch series ranging from the fastest‐growing shortest series, to the slowest growing longest series. SF‐MRCS detrending was executed in CRUST (Melvin & Briffa, 2014). RCS detrending and detrending with a horizontal line was performed in R version 4.2.1 (R Core Team, 2022) with the “dplR” package (Bunn, 2008).

All methods resulted in a lowered first‐order autocorrelation (Table A1). RCS‐detrending retained both short‐term trends and the overall positive trend in site‐level mean shoot length growth. In contrast, SF‐MRCS detrending resulted in the removal of most trends. Detrending with a horizontal line through the mean had an intermediate effect (Figures 2 and A3). Both RCS‐ and SF‐MRCS detrending reduced the mean correlation between individual branch series (rbar), signal‐to‐noise ratio, and expressed population signal (EPS; Table A1), and these methods may thus have removed some of the common climatic signal present within the series. Therefore, the series standardized with a horizontal line were used in subsequent analyses.

### 2.4 Climate data

Hourly air temperatures measured at 2 m above ground approx. 3 km southwest of the research site at ClimateBasis Disko (69.24 °N, 53.53 °W, 1 m a.s.l.; Figure 1b) covering the period 23.09.1993–31.12.2019 were downloaded from the GEM database (https://data.g‐e‐m.dk accessed on 9.3.2021; Greenland Ecosystem Monitoring, 2020). Daily mean temperatures were calculated based on these hourly measurements. Days with fewer than 16 hourly measurements were not further processed. Mean monthly temperatures were calculated based on the daily means for months with at least 20 daily means. This resulted in a mean monthly temperature series from October 1993 to December 2019, with gaps in 1993 (December), 1995 (May–July), 1997 (January, September–December), and 1998 (January). These gaps were filled, and the monthly temperature record was extended back to 1880 with temperatures from the homogenized monthly temperature dataset from Ilulissat (Vinther et al., 2006; https://crudata.uea.ac.uk/cru/data/greenland/ accessed on 19.03.2021). Monthly mean temperatures from this dataset were transferred using linear regression between this dataset and the ClimateBasis monthly means to match the level of the latter. These records are strongly correlated for each month (.79 ≤ *r* ≤ .99, *p* < .0001), with on average slightly lower temperatures recorded in Ilulissat.

Monthly precipitation sums (1901–2019) were extracted from the monthly gridded dataset CRU TS4.04 for the grid 69–69.5 °N, 53.5–53 °W (https://crudata.uea.ac.uk accessed on 14.5.2021; Harris et al., 2020). This gridded dataset was extended back to 1880 with monthly precipitation sums calculated from daily data measured at Ilulissat (1880–1991; 69.22 °N, 51.1 °W, 39 m a.s.l.) as obtained from the Global Historical Climatology Network (GHCN accessed on 10.3.2021; NOAA, 2021). Monthly precipitation sums from this dataset were transferred using linear regression between this dataset and the CRU TS4.04 monthly sums to match the level of the latter. Precipitation sums from these records are strongly correlated (.80 ≤ *r* ≤ .97, *p* < .0001) with slightly higher values recorded in Ilulissat than suggested for the grid matching the research site. The resulting monthly temperature and precipitation records span the entire period of shrub growth (1892–2013) studied here.

### 2.5 Climate‐growth model comparisons

To test the influence of seasonal climate on growth, linear mixed‐effects model analyses were performed. In these, climate–growth models were compared with a null model. Before the analyses, both the climate and standardized individual shoot length series were normalized. This was done by subtraction of the mean of the series, followed by a division with the standard deviation. Annual shoot lengths of the individual branches were included in the models as the response variable. A total of 20 climate‐growth models were compared with the null model. Mean temperatures or precipitation sums of the previous summer (June–August of the previous year), previous autumn (September–October of the previous year), early winter (November–December of the previous year), mid‐winter (January–February), late winter (March–April), winter (previous November–April), spring (May), early summer (June–July), late summer (July–August), or summer (June–August) were included as fixed effect. Autumn was defined as September–October, as frost returns in this area in September and daily mean temperatures are on average below zero, but thaw events still common in October. May is the month of snowmelt and here defined as Spring, as in April mean daily temperatures are generally well below and in June well above zero. “Early” (June–July) and “late” (July–August) summer models were included in the analyses, besides a summer (June–August) model, as June–July temperatures were found to be the main growth‐limiting factor of *C. tetragona* in the Yukon, Canada (Weijers, Pape, et al., 2018), while August temperatures were important for growth in High Arctic Svalbard (Weijers et al., 2010). Winter temperatures were found to influence shrub growth at an adjacent site (Hollesen et al., 2015). Higher winter temperatures prior to the growing season may be both beneficial, for example, through higher nutrient availability in warmer winter soils (Schimel et al., 2004), and unfavorable for growth due to the occurrence of winter warming events. As thaw events occur more frequently in early (November–December) and late (March–April) winter in the area, four different winter temperature models were included in the analyses. Furthermore, an autocorrelation structure (AR1, autoregressive process of order one) was included in each model to account for possible autocorrelation in the shoot length series. Random intercepts were included in the models for *year* to account for spatially correlated environmental conditions not captured by the fixed effects. See Table A2 for a list of the models compared in the analyses. The null model was identical to the climate models with a mean of one instead of a climate variable as fixed effect. The linear mixed‐effect model analyses were performed with the R‐package nlme v.3.1‐152 (Pinheiro et al., 2017). Maximum likelihood estimation was used for model comparison, and restricted maximum likelihood estimation was used to calculate slope estimates (Crawley, 2013). Coefficients of determination values for each model were calculated using the r.squaredGLMM function of the MuMIn package v.1.43.17 (Nakagawa & Schielzeth, 2013). The year 2014 was excluded from the analyses, as growth may not have been completed at the time of sampling in early August. The model comparison was done both at the site (across shrubs) and individual shrub level. The latter was done to test for potential differences in growth responses to climate between shrubs. To test the stability of the climate‐growth relationships over time, the model comparison was done for four different periods: the complete period (1893–2013), the most recent 50 years (1964–2013) as there was a clear increase in sample depth after 1964 (cf. Figure 2), the last complete climate period (1984–2013; that is, the last 30 years, as climate is commonly defined as the average weather over a 30‐year period), and the period excluding the last 30 years (1893–1983). Climate models that performed better than the null model were selected based on the Akaike Information Criterion (ΔAIC > 2).

### 2.6 Branch initiation and mortality frequencies

**FIGURE 3:**
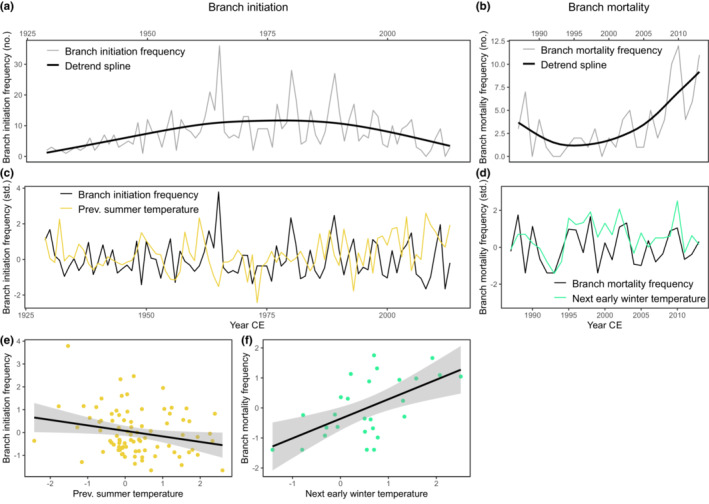
Branch initiation (a) and mortality (b) frequencies for the periods 1929–2013 and 1987–2013, respectively. The black lines represent the cubic smoothing splines (2/3 of the series length – 50 percent frequency cut‐off) with which the series were detrended. Standardized branch initiation frequency with (c) and against (e) previous summer (June–August) temperature (*r* = −.23, *p* < .05). Standardized branch mortality frequency with (d) and against (f) following early winter (November–December) temperature (*r* = .59, *p* < .002). All climate parameters shown are standardized (Z‐scores). The black lines in the scatter plots are fitted linear regression lines with 95% confidence intervals indicated in gray.

Branch initiation and mortality frequencies were measured to test for links with climate. After cross‐dating, in case the oldest part of a branch was intact with visible WMS, the year of its initiation was recorded as the first calendar year of growth. This was possible for 717 of the 764 branches measured. In addition, the last year of growth was determined for 96 branches which had an intact dead tip, with dark‐brown senesced primordial leaves. The first branch initiated in 1893. However, as for many years during the period 1893–1928 no branch initiations were recorded, branch initiation frequency analysis was restricted to the period 1929–2013. The oldest dead branch dated died in 1966 (last year of growth) or before the growing season of 1967. Only four dead branches could be dated that had died before 1987/1988. Hence, branch mortality frequency analysis covered the period 1987–2013. Both frequency series contained trends caused by either low number of old branches and a bias towards analysis of longer branch series, that is, relatively disregarding the shortest branches (cf. Figure 3a), or by senescence and organic breakdown of dead branch tips, which easily break off, and a consequent quick decline of intact dead branches back in time (cf. Figure 3b). Therefore, both series were detrended with a cubic smoothing spline 2/3 of the series length and a 50 percent frequency cut‐off (Figure 3a,b).

Both the branch initiation and mortality series as well as the climate data were normalized by subtraction of the mean, followed by division with the standard deviation, before testing for possible links. For the latter Pearson's correlation coefficients were calculated. This was done between standardized branch initiation frequency and seasonal climate (mean temperatures and precipitation sums) from early summer prior to branch initiation to late summer of the year of initiation. In addition, correlation coefficients were calculated between standardized branch mortality frequency and seasonal climate from spring of the last year of growth to late winter after the last year of growth. The same definitions of seasons were used as the climate‐growth models (see above).

### 2.7 NDVI

Bi‐weekly normalized difference vegetation index (NDVI) data for the approx. 8 × 8 km^2^ pixel incorporating the research area were extracted from the third generation of the Global Inventory Modeling and Mapping Studies (GIMMS_3g_) dataset (Pinzon & Tucker, 2014) for the period July 1981–2015. The GIMMS_3g_‐NDVI dataset is assembled from different sensors of the Advanced Very High‐Resolution Radiometers (AVHRR) satellites. It was tested whether there were positive linear trends in maximum NDVI (NDVI_max_) and mean late summer (July–August) NDVI over time with one‐sided Pearson's correlation tests. Furthermore, it was tested whether there were positive relationships between the mean standardized shoot length chronology/branch initiation on the one hand and NDVI_max_/late summer NDVI on the other. In addition, negative relationships between standardized branch mortality frequencies in the previous year and NDVI_max_/late summer NDVI were hypothesized and tested. Lastly, positive relationships between seasonal climate variables (mean temperatures and precipitation sums; the same as in the climate‐growth models) were hypothesized and tested for significance with one‐sided Pearson's correlation tests.

## 3 RESULTS

### 3.1 Shoot length series

A total of 12,528 annual growth increments were measured of 764 branches and 25 shrubs, covering 122 years over the period 1893–2014 (Figure 2, Table A1). Mean annual shoot length was 5.43 ± 1.90 mm on site level and ranged between 4.26 ± 2.10 mm and 10.20 ± 3.86 mm between shrubs (Table A3). Individual shrub series spanned between 59 (1956–2014) and 122 (1893–2014) years (Figure A2, Table A3). There were clear similarities in growth variations over time between branch series, especially within, but also between shrubs, as shown by the mean interseries correlation coefficients of 0.53 and 0.42, respectively. The Gini coefficient was 0.19 across all specimen, suggesting intermediate variability in growth, comparable to that found for the same species in the polar desert in northern Greenland (Weijers et al., 2017). Gini coefficients at the shrub level varied between 0.16 and 0.28, suggesting intermediate to high variability of growth of individual shrubs in comparison to variation in tree‐ring widths from mesic to xeric sites (Biondi & Qeadan, 2008).

### 3.2 Climate‐growth: site level

**FIGURE 4:**
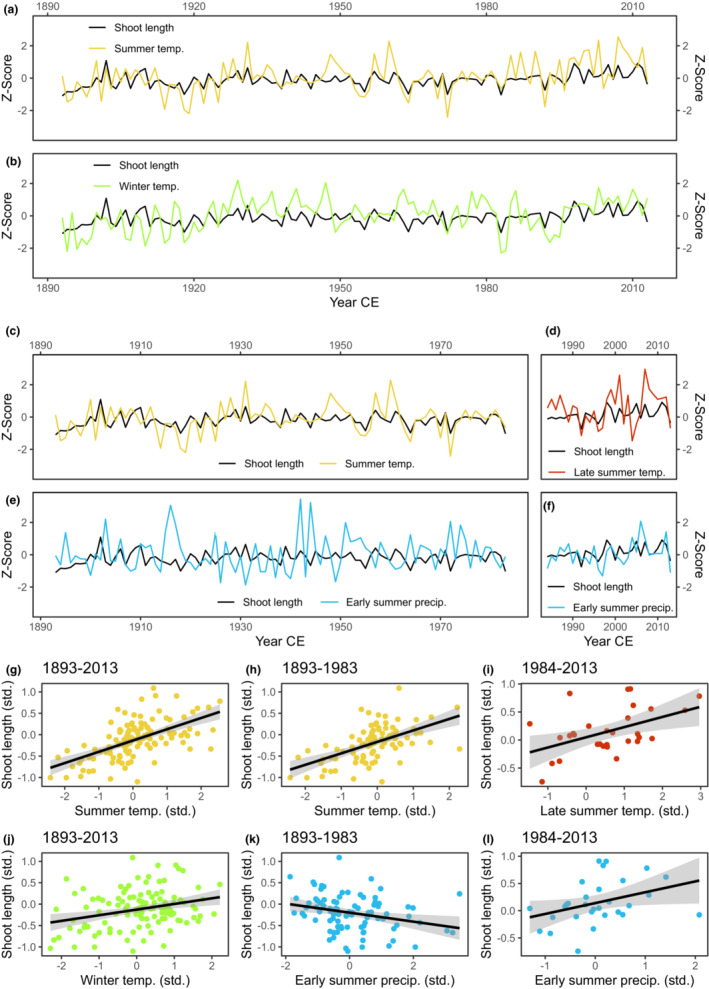
Time series (a)–(f) and scatter plots (g)–(l) of the mean site‐level shoot length with/against the fixed factor of the best climate‐growth model calculated at the site level over the periods 1893–2013 (a) and (g), 1893–1983 (c) and (h), and 1984–2013 (d) and (i); and with/against the best non‐summer temperature model over the periods 1893–2013 (b) and (j), 1893–1983 (e) and (k), and 1984–2013 (f) and (l). All parameters shown are standardized (Z‐scores). The black lines in the scatter plots are fitted linear regression lines with 95% confidence intervals indicated in gray.

The climate‐growth model comparison calculated at the site level over the entire period (1893–2013) indicated that summer temperatures were the main limiting climate factor for annual shoot length growth of *Cassiope tetragona* at the research site on Disko Island, Greenland (Table 1, Figure 4). Higher summer temperatures led to longer shoot length increments (slope: 0.25 ± 0.03). The winter temperature model (slope: 0.12 ± 0.04) was the best non‐summer temperature model at the site level over this period, suggesting a positive influence of winter temperatures on growth. The site‐level climate‐growth model comparison computed over 1964–2013 gave similar results (Table A4). The mean summer temperatures plotted together with mean site level shoot length (Figure 4a) show that summer temperature and shoot length generally fluctuated in the same direction from year‐to‐year. However, growth did not keep up with extreme summer temperatures, especially during the warm summers of the late 1940s, early 1960s and in recent decades (1980s–2010s). The model comparison at the site level for the period 1893–1983 revealed summer temperatures as the main driver of growth during this period. The early summer precipitation model was the best‐performing non‐summer temperature model for this period (∆AIC = 7.74, slope: −0.11 ± 0.04; Table 1b). The negative slope of this model indicates that before 1984 wet conditions in June–July had a negative effect on *C. tetragona* shoot length growth. The site level model comparison over 1984–2013 showed that summer temperatures were still the most limiting climate factor for growth during this period, although mean late summer (July–August) temperatures became more important than mean summer (June–August) temperatures. Notably, the slopes and ∆AIC values of the summer temperature models calculated over the recent period (1984–2013) are less steep and lower than those calculated over the complete period (1893–2013) and the period before 1984 (Table 1). The early summer precipitation model was the only non‐summer temperature model with ∆AIC > 2, suggesting that June–July precipitation sums have been a limiting factor for shoot length growth at the site level during 1984–2013. Remarkably, the slope of the early summer precipitation model changed from negative (−0.11 ± 0.04) to positive (0.20 ± 0.10) after 1983 (Table 1, Figure 4).

**TABLE 1:** Statistics of the selected climate‐growth models (∆AIC > 2) at the site level calculated over the periods 1893–2013 (a), 1893–1983 (b) and 1984–2013 (c).

Model	∆AIC	Slope ± SE	mR^2^	cR^2^
*(a) 1893–2013*				
Summer *T*	49.54	0.25 ± 0.03	0.07	0.17
Early summer *T*	44.55	0.24 ± 0.03	0.06	0.16
Late summer *T*	36.72	0.22 ± 0.03	0.06	0.17
Winter *T*	6.20	0.12 ± 0.04	0.01	0.16
Early winter *T*	5.13	0.10 ± 0.04	0.01	0.16
Prev. fall *P*	5.11	0.10 ± 0.04	0.01	0.17
Mid‐winter *T*	3.97	0.10 ± 0.04	0.01	0.16
Prev. summer *T*	3.37	0.09 ± 0.04	0.01	0.16
*(b) 1893–1983*				
Summer *T*	35.63	0.26 ± 0.04	0.05	0.11
Early summer *T*	33.87	0.25 ± 0.04	0.04	0.11
Late summer *T*	21.14	0.20 ± 0.04	0.04	0.12
Early summer *P*	7.74	−0.11 ± 0.04	0.01	0.11
Summer *P*	6.30	−0.12 ± 0.04	0.01	0.11
Prev. fall *P*	5.40	0.11 ± 0.04	0.01	0.12
Winter *T*	3.36	0.10 ± 0.04	0.01	0.11
Late winter *T*	3.04	0.10 ± 0.04	0.01	0.11
Spring *T*	2.89	0.10 ± 0.05	0.01	0.12
*(c) 1984–2013*				
Late summer *T*	5.81	0.18 ± 0.06	0.04	0.18
Summer *T*	4.45	0.17 ± 0.07	0.03	0.18
Early summer *T*	2.79	0.15 ± 0.07	0.03	0.18
Early summer *P*	2.32	0.20 ± 0.10	0.02	0.18

Abbreviations: AIC, Akaike information criterion; cR^2^, conditional coefficient of determination (proportion of variance explained by fixed and random effects); mR^2^, marginal coefficient of determination (proportion of variance explained by fixed effect); *P*, precipitation sum; prev., previous; SE, standard error; *T*, mean temperature.

### 3.3 Climate‐growth: shrub level

**FIGURE 5:**
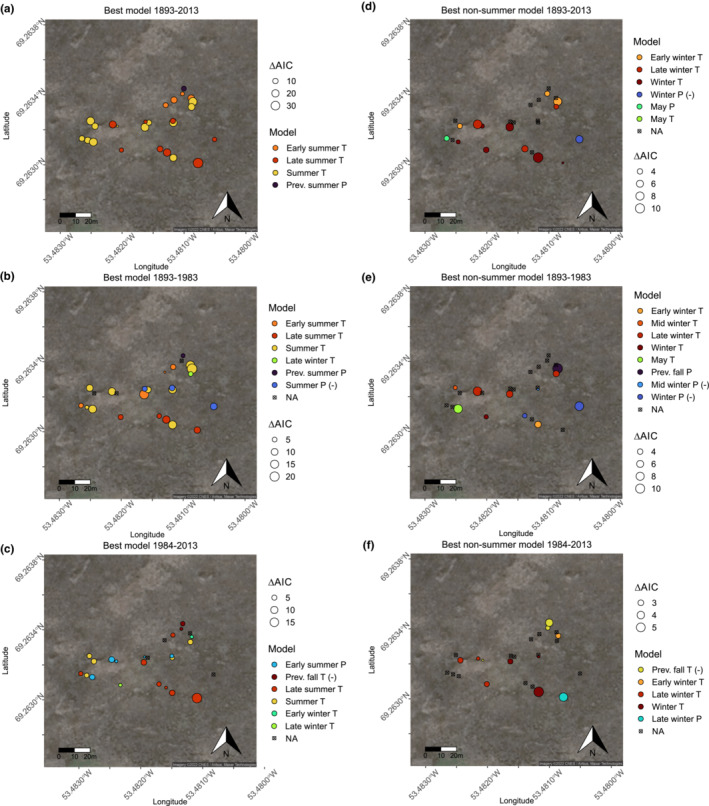
Location maps with best climate models (a)–(c) and best non‐summer climate models (d)–(f) explaining growth of individual shrubs calculated over the periods 1893–2013 (a) and (d), 1893–1983 (b) and (e), and 1984–2013 (c) and (f). Negative relations between shrub growth and climate are indicated with (−) in the model legends. The site is located on a gentle south‐southeast‐facing slope (cf. Figure 1e).

**FIGURE A4:**
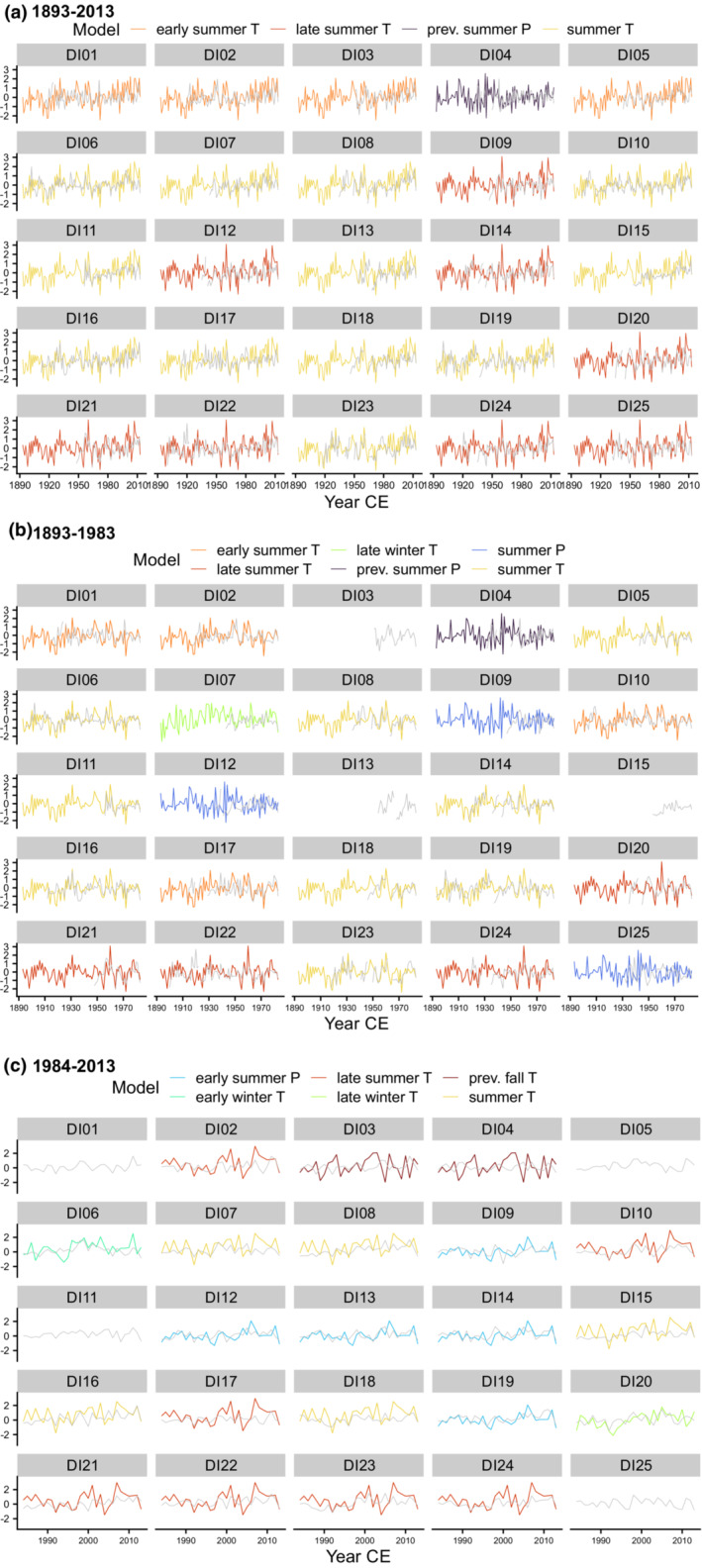
Mean shoot length (gray lines) with the fixed factor (thick coloured lines) of the best climate‐growth model for each individual shrub sample as computed over the periods 1893–2013 (a), 1893–1983 (b), and 1984–2013 (c). All parameters are shown as standardized Z‐scores.

**FIGURE A5:**
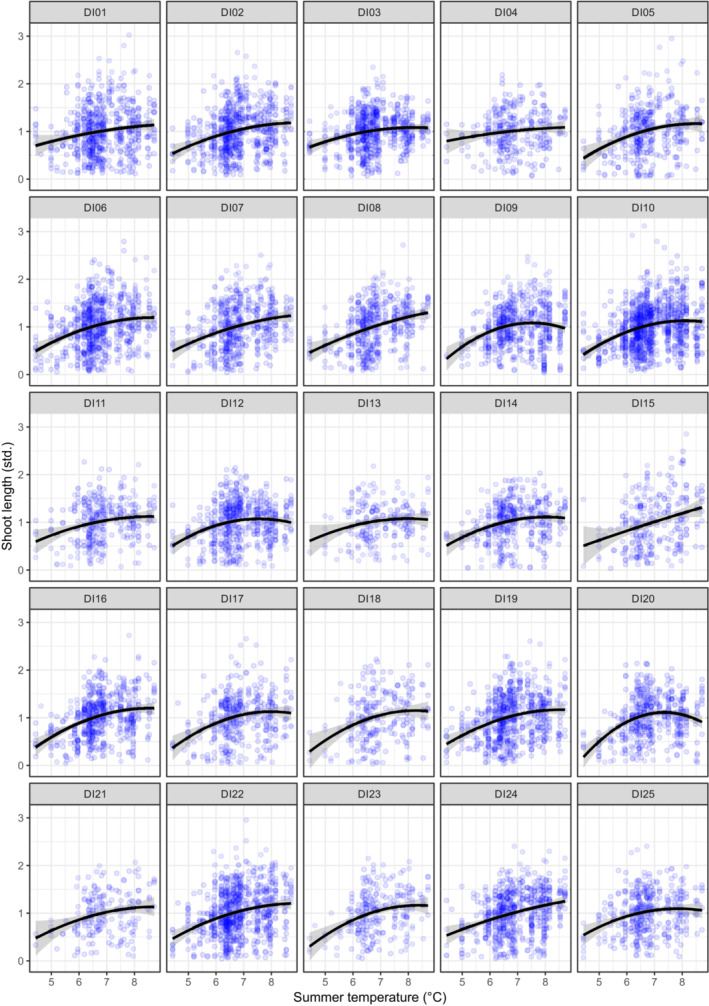
Standardized *Cassiope tetragona* shoot length growth against mean summer temperature (°C) for individual shrubs growing near Qeqertarsuaq, Disko Island, Greenland. Black lines represent polynomial functions of degree two with 95% confidence intervals in gray.

A similar picture emerged from the climate‐growth model analyses on the individual shrub level (Table A5, Figures A4 and A5). Computed over the complete period (1893–2013), all shrubs had a summer climate model as best model. The summer temperature model was the best model for 12/25, the late summer temperature model for 8/25, and the early summer temperature model for 4/25 shrubs. For one shrub, the previous summer precipitation model was the best model. All best models had a positive slope, which ranged between 0.15 and 0.38 (Table A5a). For 14/25 shrubs sampled, non‐summer climate models had a ∆AIC > 2, 12 of which were winter temperature models: growth of six shrubs responded to winter (November–April), three to early winter (November–December), and three to late winter (March–April) temperatures. All winter temperature models had a positive slope (between 0.18 and 0.33; Table A5a), suggesting a beneficial effect of higher winter temperatures preceding the growing season on shoot length development at the study site for 12 shrubs. Climate in spring (May) was found to be the best non‐summer climate model for two of the shrubs: May temperature for one and May precipitation for the other. Both these models had a positive slope. Winter precipitation had a negative impact on shoot length of one of the shrubs (DI25), which otherwise predominantly responded to late summer temperature. The model comparison calculated over 1964–2013 at the individual shrub level produced comparable results (Table A6). As seen on the site level, annual shoot length growth of many individual shrubs tracked year‐to‐year variability in summer temperatures during most years with divergence during warm extremes in recent decades, as well as earlier periods (Figures A4 and A5). There was a spatial pattern visible in the distribution of shrub response to summer climate, with shrubs responding to early summer temperatures confined to upslope positions, a late summer temperature response found mostly downslope, and a summer temperature response in between and at overlapping positions with either (Figure 5a).

The model comparison analysis at the individual shrub level for the period 1893–1983 revealed summer temperature models as the best model for 17/25 shrubs. The summer temperature model was the best model for 9/25, the late summer temperature model for 4/25, and the early summer temperature model for 4/25 shrubs. All summer temperature models had positive slopes ranging between 0.18 and 0.37. Three shrubs had summer precipitation models as best‐performing model, all with negative slopes between −0.39 and −0.26. For one shrub, the previous summer precipitation model was again the best model with a positive slope of 0.28. Another shrub had late winter temperatures as best model, also with a positive slope of 0.28. Three shrubs were insensitive to climate (Table A5). Over this period (1893–1983), non‐summer climate models had a ∆AIC > 2 for 11/25 shrubs, six of which were winter temperature models: growth of one shrub responded to winter (November–April), one to early winter (November–December), one to mid‐winter (January–February) and three to late winter (March–April) temperatures. All winter temperature models had a positive slope ranging between 0.20 and 0.33 (Table A5a). Winter precipitation was found to be the best‐performing non‐summer model for three shrubs: one shrub responded to mid‐winter and two to winter precipitation with slopes between −0.29 and −0.20, suggesting a negative impact of snow on growth of these shrubs. For one shrub previous autumn (September–October) precipitation was the best non‐summer model (slope 0.22), and for one shrub spring temperatures (slope 0.26; Table A5b). The spatial pattern in the distribution of shrub responses to summer climate over this period (1893–1983; Figure 5b) was similar to that observed for the complete period (Figure 5a) with shrubs which responded to early summer temperatures mainly restricted to upslope positions, those which responded to late summer temperatures located further downslope, and those responsive to summer temperatures located in between and at overlapping positions. The shrubs with a positive response to previous summer precipitation (Figure 5b) or prev. fall precipitation (Figure 5e) were located upslope, while those with a negative response to summer precipitation (Figure 5c) or winter precipitation (Figure 5e) were located further downslope.

Summer temperature models best‐explained growth for 12/25, that is, less than half of the shrubs, when the analysis was performed over the period 1984–2013 (Table A5b, Figure A4b). Shoot length growth of seven shrubs responded to late summer (July–August) temperature and that of five to summer (June–August) temperature. These summer temperature models had a positive slope, which ranged between 0.17 and 0.34 (Table A5b). For 5/25 shrubs, the early summer precipitation model was the best model (slope between 0.25 and 0.38). For 4/25 shrubs, a non‐summer climate model was the best model. For two shrubs, early or late winter temperature models best‐explained shoot length growth. Both models had a positive slope. Two other shrubs showed a negative response to previous fall temperatures (Table A5c). Four of the 25 shrubs did not respond to climate over this period. Over 1984–2013, non‐summer climate models had a ∆AIC > 2 for 11/25 shrubs, 7 of which were winter temperature models: one early (prev. November–prev. December), three late (March–April), and three winter (prev. November–April) temperature models, all with a positive slope (Table A5b). Three shrubs showed a negative response to previous fall temperatures. In contrast to the calculations over 1893–2013 and 1893–1983, in the recent period (1984–2013) late winter precipitation had a positive impact on shoot length of one of the shrubs (DI24), which pre‐dominantly responded to late summer temperature. Shrubs with non‐summer models as best model and non‐responsive shrubs were generally positioned further upslope (Figure 5c), as were shrubs with previous fall temperature as best non‐summer model (Figure 5f).

### 3.4 Branch initiation and mortality

Over the period studied (1929–2013), branch initiation frequency was negatively correlated with mean summer temperatures in the year prior to branch initiation (*r* = −.23, *p* < .05; Figure 3c), that is, warm summers were linked with a lower amount of new branches formed in the year after. Over the period 1987–2013, branch mortality frequency was positively correlated with mean early winter temperatures following the growing season in the year of death (*r* = .59, *p* < .002; Figure 3d) and with mean temperatures in spring prior to the growing season (*r* = .50, *p* < .01). Higher temperatures before and after the growing season were thus related with higher branch mortality.

### 3.5 NDVI

**FIGURE 6:**
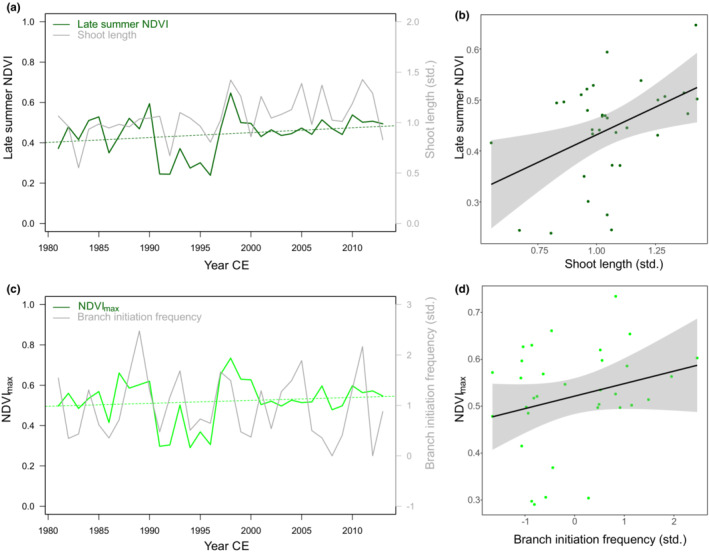
Mean late summer (July–August) NDVI over 1981–2013 with (a) and against (b) standardized mean shoot length site‐chronology (*r* = .44, *p* < .01). There was a positive trend in late summer NDVI over this period (*r* = .24, *p* < .1). Maximum NDVI with (c) and against (d) standardized branch initiation frequency and (*r* = .26, *p* < .1). The black lines in (b) and (d) are fitted linear regression lines with 95% confidence intervals indicated in gray.

Standardized mean site‐level shoot length growth was positively correlated with late summer (July–August) NDVI (*r* = .44, *p* < .01, Figure 6a,b) as well as NDVI_max_ (*r* = .40, *p* < .05). There was a weak link between standardized branch initiation and NDVI_max_ (*r* = .26, *p* < .1; Figure 6c,d). There were no negative correlations between standardized branch mortality frequency and NDVI in the research area. There was a slight positive trend in late summer NDVI over the period 1981–2013 (*r* = .24, *p* < .1; Figure 6a), but not in NDVI_max_.

Late summer NDVI and NDVI_max_ were positively related to summer and, notably, winter temperatures (Table 2). Furthermore, there were positive relationships between late summer NDVI and spring precipitation sums, and weakly, late winter precipitation sums (Table 2).

**TABLE 2:** Pearson's correlation coefficients between climate variables and late summer (July–August) NDVI/maximum NDVI over the research area. Only coefficients with *p* < .1 are shown. *^.^p* < .1, *p < .5, **p < .01.

Variable	Late summer NDVI	NDVI_max_
Summer *T*	0.47**	0.48**
Early summer *T*	0.45**	0.44**
Late summer *T*	0.40*	0.35*
Late winter *T*	0.32*	0.25^.^
Spring *P*	0.30*	–
Winter *T*	0.30*	0.26^.^
Late winter *P*	0.26^.^	–
Early winter *T*	0.23^.^	–

## 4 DISCUSSION

### 4.1 Temperature‐growth decoupling

The shoot length growth response to summer temperatures of *Cassiope tetragona* at the site level over the entire period studied (1893–2013) and over the period 1893–1983 can be classified as highly sensitive (∆AIC = 49.54 and ∆AIC = 35.63, respectively; cf. Myers‐Smith et al., 2015). However, in recent decades (1984–2013) this sensitivity can be categorized as low (∆AIC = 5.78) and it was completely absent for over half of the individuals studied, as shown by the analyses on individual shrub level. While late summer (July–August) temperatures were the most limiting factor for growth at the site level over the period 1984–2013, early summer precipitation was identified as an important co‐driver of growth at the site level, with a steeper slope than that of the summer temperature models, and as the most limiting factor for 20 percent of the individuals studied here. Notably, the observed link between summer precipitation and growth at the site level was negative until 1984, but positive afterward. Likewise, the observed links between either summer or winter precipitation and individual shrub growth were predominantly negative before 1984, but exclusively positive since. These findings suggest a decreased temperature and increased moisture sensitivity of *Cassiope tetragona* growth under climate warming in the already relatively warm Low Arctic. A similar divergence between growing season temperatures and evergreen dwarf shrub growth has recently been demonstrated in the French Alps for the species *Rhododendron ferrugineum* (Francon et al., 2021). Moreover, a similar reduction in temperature sensitivity, possibly related to increased drought stress, has been observed for tree growth at high northern latitudes in recent (see, e.g., D'Arrigo et al., 2008) as well as past warm decades (e.g., Schneider et al., 2014) and is known as the “Divergence Problem.”

**FIGURE A6:**
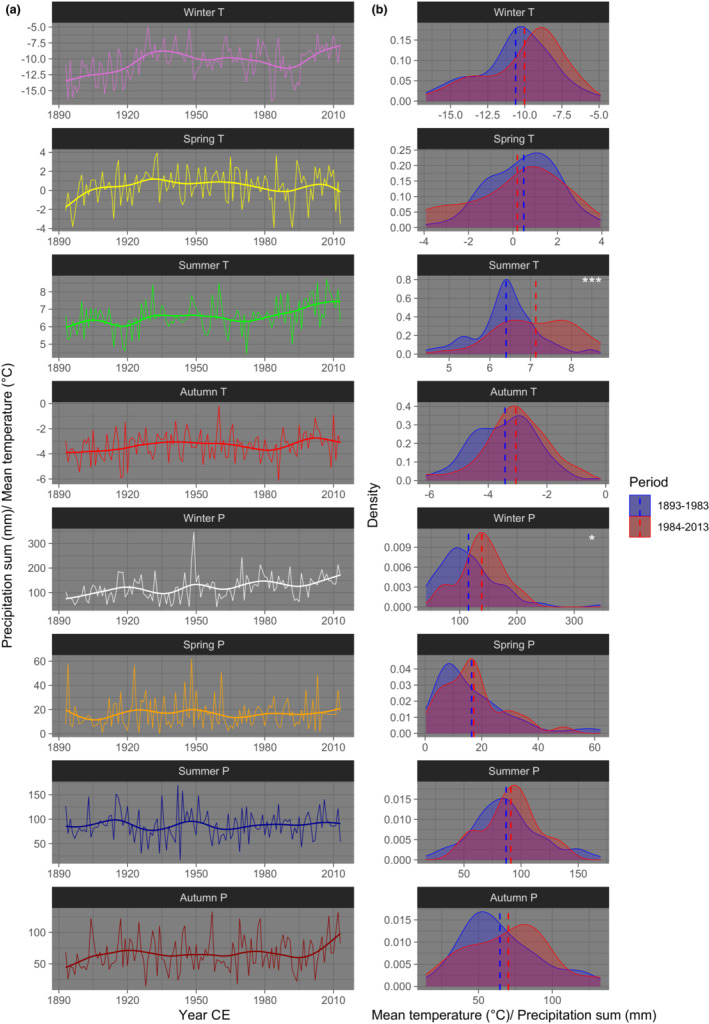
Seasonal mean temperatures and precipitation sums over the period 1893–2013 (a). Thick lines are 32‐year cubic smoothing splines with a 50 percent frequency cut‐off, indicating trends in the series. Probability density functions for mean seasonal temperatures and precipitation sums for the periods 1893–1983 (blue) and 1984–2013 (red) (b). Dashed lines show mean values. Asterisks indicate significant differences between means (two‐sided *t*‐test). *p < .05, ***p < .001, *P*, precipitation sum; *T*, mean temperature.

The observed lower sensitivity of shrub growth to summer temperature in recent decades was probably caused by the on average warmer summers during this period (7.13°C in 1984–2013 vs. 6.40°C in 1893–1983, *p* < .001; Figure A6). This, in combination with the observed non‐linear growth response to summer temperatures, with a smaller, lack of, or even negative response to mean summer temperatures exceeding a threshold of approx. 7°C (Figure A5). Although the shrubs were capable of maintaining high levels of shoot length growth in summers with mean temperatures exceeding this threshold, other factors than summer temperature became more limiting for the majority of the shrubs studied. Interestingly, Gamm et al. (2018) reported a weakening in temperature‐growth relationships in *Betula nana* and *Salix glauca* shrubs in more continental Greenland, 272 km southeast of Disko Island, when summer (there: May–August) temperatures passed a threshold of approx. 7°C. A similar non‐linear response of *C. tetragona* shoot length growth was observed over a gradient of growing degree‐day sums (GDD_5_; cumulative daily mean temperatures above 5°C) across three sites, two in High Arctic Svalbard, one in Sub‐Arctic Sweden (Weijers, Wagner‐Cremer, et al., 2013). It is, therefore, likely that this evergreen dwarf shrub species will not be able to respond with increased growth rates to warmer summers in the Low and Sub‐Arctic under future global warming. This may lower its competitiveness, especially for light, in relation to taller deciduous shrub species, which are migrating into tundra ecosystems (Bjorkman et al., 2018). However, growth of such species has been shown to be less sensitive to warming at drier sites than at moister sites (Ackerman et al., 2017; Myers‐Smith et al., 2015). The dwarf shrub *C. tetragona*, on the other hand, is restricted to relatively dry soils (Rune, 2011) and adapted to dry conditions with its xeromorphic leaves (Havström et al., 1993). Therefore, it may remain competitive in drier habitats.

In this study, a positive influence of summer precipitation on growth of *C. tetragona* was detected for some of the shrub individuals over the period 1984–2013. Summer precipitation was observed to be a co‐driver of *C. tetragona* growth for one of four sites on Ellesmere Island in High Arctic Canada (Myers‐Smith et al., 2015). However, a similar influence had not yet been observed for this species at other sites. Not at relatively warm tundra sites in the Sub‐Arctic (Weijers et al., 2012; Weijers, Pape, et al., 2018), nor in the High Arctic (Rayback & Henry, 2006; Weijers et al., 2010, 2012), including the northernmost polar desert in northern Greenland (Weijers et al., 2017). In addition, *C. tetragona* growth did not respond to experimentally doubled summer precipitation in High Arctic Svalbard (Weijers, Auliaherliaty, et al., 2013). The reason for a lack of detection of a summer precipitation signal in other Arctic sites could be (micro)site‐specific. Soil moisture availability may be largely decoupled from summer precipitation and instead determined by downslope meltwater run‐off, active layer thaw, and low evapotranspiration rates in the High Arctic (Weijers et al., 2017). At Arctic‐alpine sites with late snow cover, where the species is found (Weijers, Pape, et al., 2018), snowmelt likely provides sufficiently high soil moisture levels throughout the growing season. In addition, at other sites, climate‐growth relationships have not been assessed at the individual level and sensitivity to precipitation may not only be site‐specific but also micro‐site specific, due to, for example, greater soil moisture availability in small depressions or snow accumulation behind rocks. Remarkably, two of the shrubs studied here (DI15 and DI16) grew near rocks and their growth remained sensitive to summer temperatures in recent decades (Figure A4), perhaps related to micro‐site conditions. Furthermore, climate‐growth links have mostly solely been studied over longer periods, and did not focus on the recent warm period. However, growth of the deciduous dwarf shrub *Betula nana* in the northeastern Siberian tundra was shown to be sensitive to summer precipitation in warm, but not in cool summers (Li et al., 2016). In addition, shoot length growth of *C. fastigiata* was detected to be sensitive to moisture availability in the tree‐line ecotone (Rayback et al., 2017) and growth of *Juniperus indica* shrubs was found to be driven by early growing season precipitation (Pandey et al., 2020), both in the central Himalayas of Nepal. Moreover, low amounts of summer precipitation as a co‐determinant of drought conditions have been shown to indirectly drive declining deciduous shrub growth at drier sites, while summer temperature drives increasing deciduous shrub growth at wetter sites in the Arctic (Buchwal et al., 2020).

### 4.2 Influence of winter temperatures

**FIGURE A7:**
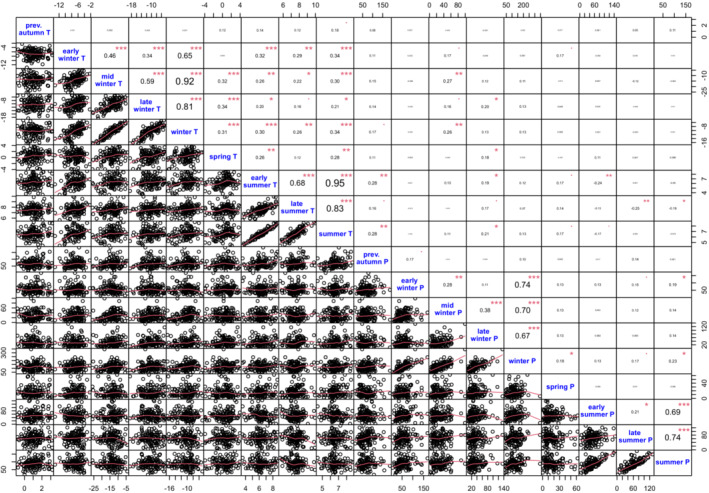
Correlation matrix over the study period 1893–2013 for the climate parameters used as fixed effects in the linear mixed effect model comparison. Values in the panels are Pearson's correlation coefficients. Scatterplots give the relationships between the parameters. Significance levels are indicated as: *^.^p* < .1, *p < .05, **p < .01, ***p < .001.

**FIGURE A8:**
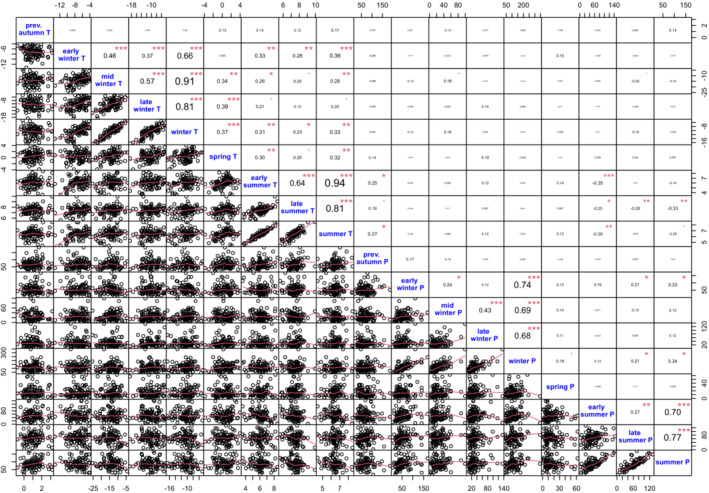
Correlation matrix over the study period 1893–1983 for the climate parameters used as fixed effects in the linear mixed effect model comparison. Values in the panels are Pearson's correlation coefficients. Scatterplots give the relationships between the parameters. Significance levels are indicated as: *^.^p* < .1, *p < .05, **p < .01, ***p < .001.

**FIGURE A9:**
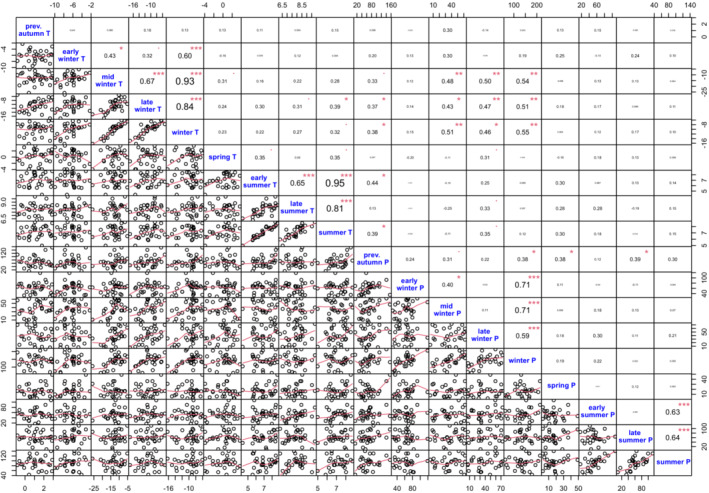
Correlation matrix over the study period 1984–2013 for the climate parameters used as fixed effects in the linear mixed effect model comparison. Values in the panels are Pearson's correlation coefficients. Scatterplots give the relationships between the parameters. Significance levels are indicated as: *^.^p* < .1, *p < .05, **p < .01, ***p < .001.

Winter temperatures preceding the growing season were observed to be the main co‐driver of *C. tetragona* growth at the site level and for 11/25 shrubs studied over the period 1893–2013. Over the period 1893–1983, it was the best non‐summer climate‐growth model for six shrubs, and the best model explaining growth for one. In recent decades, it was found to be the best non‐summer climate‐growth model for five shrubs and the main climatic driver for two. Notably, a similar positive effect of warm winters on dwarf shrub growth was observed for the deciduous dwarf shrub *Betula nana* at an adjacent site on Disko Island (Hollesen et al., 2015). In their study, the positive effect of winter temperatures on growth was interpreted as a result of warmer winters and related spring air temperatures causing snow to melt out earlier, allowing soils to drain and warm faster. This then results in longer growing seasons, leading to increased growth. For *C. tetragona* at the study site this mechanism is less plausible, as early summer temperatures were the main climatic driver of only four shrubs (1893–2013 and 1893–1983) and of none in recent decades. This suggests that this species may not benefit from earlier snow melt‐out, which is in line with the observation that *C. tetragona* growth benefits from warmer Julys, but not from longer growing seasons near its European southern distribution limit in Abisko, Sweden (Weijers, Wagner‐Cremer, et al., 2013). An alternative explanation could be grounded on the fact that winter air temperatures are positively related to winter precipitation sums in Disko Island (Figures A7, A8, A9), although winter precipitation was negatively linked to growth of some of the shrubs in this study before 1984 (Figure 5e). In warmer winters, an increase in winter snow precipitation may result in a deeper snowpack and higher winter soil temperatures due to insulation. Moreover, in recent decades winter soil temperatures have been directly coupled with winter air temperatures in the area (Hollesen et al., 2015). Warmer winter soil temperatures may be linked to higher microbial activity in winter (Schimel et al., 2004) and result in greater nutrient availability in the subsequent growing season (Natali et al., 2012) and hence support increased shrub growth. In addition, warm winters are negatively linked with sea ice extent in the Disko Bay area (Hollesen et al., 2015) and earlier sea ice retreat leads to warmer summers (Buchwal et al., 2020). This may explain the positive correlation between winter and summer temperatures at the site (Figures A7, A8, A9) and partly or alternatively explain the observed association between winter temperatures and *C. tetragona* growth. A similar possible link between sea‐ice conditions prior to the growing season, summer temperatures, and *C. tetragona* growth has previously been suggested for a site in northern Greenland (Weijers et al., 2017).

### 4.3 Micro‐topography

Shrubs responsive to early summer temperatures were restricted to upslope positions, while those responsive to late summer temperatures were found further downslope. This pattern may be related to the redistribution of snow by wind with accumulation of snow according to topography (Erickson et al., 2005). In the study area, winter winds blow mainly from northern direction (Blok et al., 2016), and the downslope positions may thus be more wind‐sheltered and be covered in a deeper snow layer. Upslope positions would then be snow‐free earlier, which may explain the responsiveness to early summer temperatures found there. Upslope soils will drain and warm faster and are thus drier, which could be the reason for the response to previous summer precipitation of the northernmost shrub in this study. Remarkably, some of the shrubs further downslope responded negatively to summer and/or winter precipitation before 1984 (Figure 5). This could also explain the negative response to previous autumn temperatures over the period 1984–2013 of some of the upslope shrubs, with warm dry autumns resulting in lower soil moisture availability in the following growing season. A negative influence of autumn temperatures was recently also observed for shoot length growth of the related boreal shrub *Empetrum nigrum*, growing near its southern lowland distribution limit in northern Germany (Hein et al., 2021). Over the period 1984–2013, non‐responsive shrubs were mostly found at upslope positions and shrubs responsive to early summer precipitation sums mostly mid‐slope. Shrubs at downslope positions were mostly still responsive to late summer temperatures. This is in line with the hypothetical gradient in snow depth.

### 4.4 Branching and branch mortality

**FIGURE A10:**
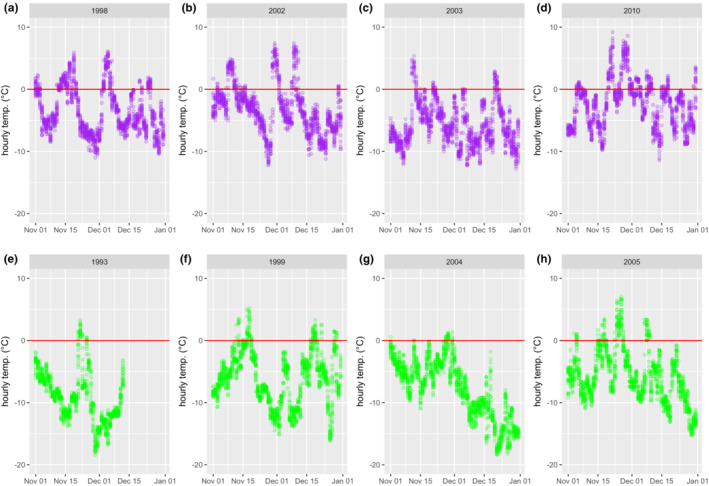
Hourly air temperatures measured at 2 m above ground approx. 3 km southwest of the research site at ClimateBasis Disko (69.24 °N, 53.53 °W, 1 m a.s.l.; https://data.g‐e‐m.dk). For the four years with high standardized branch mortality frequencies (>mean + one standard deviation) in purple (a–d) and for the four years with the lowest standardized branch mortality rates in green (e,f). Standardized branch mortality frequency was lower than the mean minus one standard deviation in 1993 and 1999. The hourly temperature dataset starts on 23.09.1993, but data is missing for the second half of December 1993.

Warm early winters (November–December) after the growing season may have led to higher branch mortality rates. In years with high branch mortality, hourly temperatures were in general more frequently above freezing point in November–December than in years with low branch mortality (Figure A10). Such winter warming events can cause reduced snow cover leaving the plants vulnerable to subsequent freezing damage and dieback (Bokhorst et al., 2009, 2016), as observed for *C. tetragona* in Svalbard (Bjerke et al., 2017). Moreover, winter warming often coincides with rain‐on‐snow events (Le Moullec et al., 2020), the frequency of which has increased in Greenland in recent decades (Niwano et al., 2021). Such events can lead to encasement of plants in ground ice, which increases branch mortality in *C. tetragona*, as has been shown experimentally in Svalbard (Milner et al., 2016). In the same icing experiment, it was found that shoot length of surviving branches increases after icing treatment. This may offer an additional or alternative explanation for the positive influence of winter temperatures on *C. tetragona* growth observed here.

Warm springs (Mays) prior to the growing season may have resulted in increased branch mortality, following a similar mechanism. Spring warming may lead to an advanced start of the growing season. This can leave plants exposed to late frost, which can result in damage to soft tissues and reduced growth, as was observed in the evergreen shrubs *Empetrum nigrum* ssp. *hermaphroditum*, at a near tree‐line site in the Central Norwegian Scandes (Weijers, Beckers, & Löffler, 2018) and *Rhododendron ferrugineum* at a low‐alpine site in the French Alps (Francon et al., 2020).

Branching occurred more often after years with cool summers than after years with warm summers. There was also a negative correlation between shoot length growth in the previous year and branch initiation frequency (*r* = −.21, *p* < .05). Hence, when shoot length growth in the previous growing season is below average, perhaps due to a cool summer, *C. tetragona* shrubs tend to invest more in new branches to increase or maintain its photosynthetic capacity. Campioli et al. (2012) observed an increase in the number of branches in *C. tetragona* shrubs in response to experimental shading. Similarly, the shrubs seem to form more side branches after recent stress, such as damage to branch tips (own observation). Dead or damaged *C. tetragona* shoot tips with a cluster of new branches are also a remarkable feature of shrubs exposed to experimental icing (Milner et al., 2016). Likewise, browsing by ptarmigan and moose leads to increased production of vegetative shoots in the deciduous tall shrub *Salix alaxensis* in northern Alaska (Christie et al., 2014). The capacity of making new branches, sometimes, though rarely, on decades‐old *C. tetragona* stem segments, may be related to their presence at the basal part of annual shoot increments (Warming, 1908), that is, near WMS, which consist of green meristem tissue. *C. tetragona* may thus be well‐adapted to and capable of recovery from branch dieback caused by winter warming events, as its primordia are often protected under old leaves at lower stem positions.

### 4.5 Correspondence between growth, branching, and NDVI

Annual variation in *C. tetragona* shoot‐length growth and branching frequency positively related to fluctuations in satellite‐observed vegetation greenness over the research area. This suggests that there is, at least to some degree, correspondence in annual variation in community‐level vascular plant biomass. This is in line with Milner et al. (2018), who found that annual growth of this species represents annual above‐ground tundra vegetation productivity at the local scale. Moreover, the same climate variables, that is, summer and winter temperatures, as observed for the evergreen dwarf shrub *C. tetragona* drove year‐to‐year satellite‐observed variation in greenness. The same climate parameters determine growth rates of the co‐dominant deciduous dwarf shrub *B. nana* in the research area in the Blæsedalen valley on Disko Island (Hollesen et al., 2015). Such similarity in climatic drivers of growth patterns between shrub species with contrasting life strategies and NDVI was earlier observed over Arctic‐alpine areas in northwest North America (Weijers, Pape, et al., 2018). The lack of a link between branch mortality and NDVI may be due to increased branching after winter warming events (Milner et al., 2016). Satellite‐observed vegetation productivity in the research area did not correlate with early summer precipitation, but there were positive relations with late winter and spring precipitation. Growth and productivity of the shrub‐dominated vegetation in the Blæsedalen valley may thus be increasingly moisture‐limited. It is, however, not in decline, in contrast to *B. nana* and *S. glauca* in a warmer and drier area on the mainland of Greenland (Gamm et al., 2018), approx. 270 km south‐southeast of Disko Island. While there was a slight increasing trend in late summer NDVI, there was no trend in NDVI_max_, which is in line with the widespread vegetation stability observed over large parts of the Arctic (Callaghan et al., 2021).

## 5 CONCLUSIONS

Since at least the late 19th century, summer temperatures have been the main climatic driver of growth of the evergreen dwarf shrub *Cassiope tetragona* at the Low‐Arctic research site near Qeqertarsuaq on the southern tip of Disko Island, western Greenland. Winter temperatures have been a co‐driver of shrub growth. In recent and past warm decades, however, shrub growth has diverged from summer temperatures. For some individual shrubs, apparently mostly those growing at positions with lower soil moisture availability as determined by micro‐topography, early summer precipitation has become the main driver during the recent climatic period (1984–2013), while over half of the shrubs studied became insensitive to summer temperatures. The correspondence between climatic drivers, *C. tetragona* growth and branch initiation frequency, and satellite‐observed vegetation productivity (NDVI), suggests that the climate‐growth observations for *C. tetragona* at least partly apply to the entire shrub‐dominated vegetation in the research area. Winter warming events are likely the most prevalent cause of branch mortality in *C. tetragona*. Increased branching after years with poor growth and cooler than average summers seems related to stress and may enable the shrub to overcome damage, for example, caused by winter warming events.

These findings suggest that erect dwarf‐shrub tundra in the Low Arctic has already and will likely become decreasingly temperature‐ and increasingly moisture‐limited under future warming and that, although winter warming supports shrub growth, increased frequency of extreme winter warming events will likely lead to increased branch mortality and damage the dwarf‐shrub tundra. The observed divergence between summer temperatures and shrub growth during warm phases and the contrasting, potentially balancing effects of warmer winter temperatures may offer a mechanistic explanation for the still widespread stability of Arctic tundra vegetation.

## AUTHOR CONTRIBUTIONS

**Stef Weijers:** Conceptualization (equal); data curation (equal); formal analysis (equal); funding acquisition (equal); investigation (equal); methodology (equal); project administration (equal); resources (equal); software (equal); supervision (equal); validation (equal); visualization (equal); writing – original draft (equal); writing – review and editing (equal).

## CONFLICT OF INTEREST

There is no conflict of interest.

## Data Availability

Shoot length and branch demography data can be found at https://doi.org/10.5061/dryad.rbnzs7hf4; Hourly air temperatures from ClimateBasis Disko: GEM database: https://data.g‐e‐m.dk; Ilulissat homogenized monthly temperatures: https://crudata.uea.ac.uk/cru/data/greenland/; CRU TS4.04: https://crudata.uea.ac.uk; Ilulissat monthly precipitation sums: Global Historical Climatology Network (GHCN): https://www.ncei.noaa.gov; Bi‐weekly normalized difference vegetation index (NDVI) data from the Global Inventory Modeling and Mapping Studies (GIMMS3g) dataset: http://poles.tpdc.ac.cn/en/data/9775f2b4‐7370‐4e5e‐a537‐3482c9a83d88/.

## APPENDIX 1

**TABLE A1:** Descriptive statistics of the raw and detrended *Cassiope tetragona* site chronologies constructed with three different standardization methods.

	Raw	Hor	RCS	SF‐MRCS
Period	1893–2014	1893–2014	1893–2014	1893–2014
No. years	122	122	122	122
Mean	5.43 (mm)	0.93	0.89	0.96
s.d.	1.90	0.23	0.23	0.19
Gini	0.19	0.13	0.14	0.11
ar1	0.69	0.28	0.46	0.11
No. branches/shrubs	764/25	764/25	764/25	764/25
rbar.tot	0.43	0.43	0.28	0.28
rbar.ws	0.54	0.57	0.36	0.37
rbar.bs	0.42	0.42	0.28	0.28
rbar.eff	0.53	0.53	0.39	0.39
EPS	0.95	0.95	0.92	0.92
SNR	28.45	28.45	15.92	16.16
*n*, SSS > 0.85	7	7	9	9
period, SSS > 0.85	1914–2014	1914–2014	1915–2014	1915–2014

Abbreviations: ar1, first‐order autocorrelation; bs, between shrubs; eff, effective; EPS, expressed population signal; gini, gini coefficient; Hor, horizontal line through the mean; rbar, mean interseries correlation; RCS, regional curve standardization; s.d., standard deviation; SF‐MRCS, signal‐free multiple regional curve standardization; SNR, signal to noise ratio; SSS, subsample signal strength; tot, total; ws, within shrubs.

**TABLE A2:** Predictors used as fixed effects in the linear mixed effect model comparison analyses. Shoot length was the response variable. In each model *year* was included as random effect on the intercept. In addition, an autocorrelation structure (AR1, autoregressive process of order one) was included in each model.

Model	Predictor	Random effect (intercept)	Autocorrelation structure
1	Prev. summer *T*	Year	AR1
2	Prev. autumn *T*	Year	AR1
3	Early winter *T*	Year	AR1
4	Mid‐winter *T*	Year	AR1
5	Late winter *T*	Year	AR1
6	Winter *T*	Year	AR1
7	Spring *T*	Year	AR1
8	Early summer *T*	Year	AR1
9	Late summer *T*	Year	AR1
10	Summer *T*	Year	AR1
11	Prev. summer *P*	Year	AR1
12	Prev. autumn *P*	Year	AR1
13	Early winter *P*	Year	AR1
14	Mid‐winter *P*	Year	AR1
15	Late winter *P*	Year	AR1
16	Winter *P*	Year	AR1
17	Spring *P*	Year	AR1
18	Early summer *P*	Year	AR1
19	Late summer *P*	Year	AR1
20	Summer *P*	Year	AR1

**TABLE A3:** Descriptive statistics of the raw shrub chronologies.

Shrub	First year	Last year	No. years	Mean ± s.d.	Gini	ar1
DI01	1915	2014	100	4.41 ± 1.71	0.21	0.50
DI02	1918	2014	97	4.26 ± 2.10	0.27	0.69
DI03	1951	2014	64	5.23 ± 1.67	0.18	0.66
DI04	1932	2014	83	4.82 ± 1.85	0.22	0.45
DI05	1943	2014	72	5.84 ± 1.70	0.16	0.47
DI06	1895	2014	120	5.40 ± 2.00	0.21	0.58
DI07	1943	2014	72	5.47 ± 1.96	0.20	0.55
DI08	1934	2014	81	6.77 ± 3.25	0.27	0.73
DI09	1947	2014	68	6.89 ± 2.23	0.18	0.35
DI10	1900	2014	115	5.41 ± 1.80	0.18	0.64
DI11	1956	2014	59	7.51 ± 2.18	0.16	0.52
DI12	1935	2014	80	6.41 ± 2.04	0.17	0.12
DI13	1954	2014	61	6.67 ± 2.36	0.20	0.46
DI14	1918	2014	97	6.79 ± 2.38	0.20	0.44
DI15	1953	2014	62	8.98 ± 3.48	0.21	0.58
DI16	1912	2014	103	8.15 ± 3.70	0.24	0.60
DI17	1914	2014	101	5.34 ± 2.67	0.28	0.63
DI18	1946	2014	69	6.87 ± 3.08	0.25	0.64
DI19	1893	2014	122	6.38 ± 2.82	0.25	0.62
DI20	1935	2014	80	10.20 ± 3.86	0.21	0.39
DI21	1948	2014	67	5.43 ± 2.05	0.21	0.60
DI22	1900	2014	115	5.18 ± 2.11	0.22	0.55
DI23	1918	2014	97	8.51 ± 2.94	0.19	0.52
DI24	1919	2014	96	7.58 ± 2.60	0.19	0.55
DI25	1932	2014	83	7.76 ± 2.37	0.17	0.44

Abbreviations: ar1, first‐order autocorrelation; gini, gini coefficient; s.d., standard deviation.

**TABLE A4:** Statistics of the selected climate‐growth models (∆AIC > 2) at the site level calculated over the period 1964–2013.

1964–2013				
Model	∆AIC	Slope ± SE	mR^2^	cR^2^
Summer *T*	24.44	0.27 ± 0.05	0.08	0.20
Early summer *T*	20.88	0.26 ± 0.05	0.07	0.20
Late summer *T*	20.79	0.25 ± 0.05	0.07	0.20
Winter *T*	3.26	0.15 ± 0.06	0.02	0.20
Early winter *T*	3.10	0.14 ± 0.06	0.02	0.20
Prev. summer *P*	3.01	0.18 ± 0.08	0.02	0.20

Abbreviations: AIC, Akaike Information Criterion; cR^2^, conditional coefficient of determination (proportion of variance explained by the fixed and random effects); mR^2^, marginal coefficient of determination (proportion of variance explained by the fixed effect); *P*, precipitation sum; prev., previous; SE, standard error; *T*, mean temperature.

**TABLE A5:** Statistics of the selected climate‐growth models (∆AIC > 2) for individual shrubs calculated over the periods 1893–2013 (a), 1893–1983 (b), and 1984–2013 (c).

Shrub	Model	∆AIC	Slope ± SE	mR^2^	cR^2^
*(a) 1893–2013*					
DI01	Early summer *T*	8.55	0.19 ± 0.06	0.04	0.25
	Summer *T*	5.42	0.16 ± 0.06	0.03	0.25
DI02	Early summer *T*	13.78	0.25 ± 0.06	0.07	0.32
	Summer *T*	13.49	0.25 ± 0.06	0.06	0.32
	Late summer *T*	7.1	0.19 ± 0.06	0.04	0.31
DI03	Early summer *T*	5.21	0.20 ± 0.07	0.04	0.33
	Summer *T*	4.51	0.19 ± 0.07	0.04	0.33
	Early winter *T*	3.65	0.19 ± 0.08	0.04	0.33
	Winter *T*	3.21	0.19 ± 0.08	0.04	0.33
	Mid winter *T*	2.12	0.17 ± 0.08	0.03	0.33
DI04	Prev. summer *P*	8.09	0.25 ± 0.08	0.04	0.04
	Early summer *T*	3.17	0.14 ± 0.06	0.02	0.02
	Summer *T*	2.33	0.13 ± 0.06	0.02	0.02
DI05	Early summer *T*	15.9	0.27 ± 0.06	0.08	0.22
	Summer *T*	15.82	0.28 ± 0.06	0.08	0.22
	Late summer *T*	10.92	0.23 ± 0.06	0.06	0.23
	Prev. summer *T*	3.8	0.16 ± 0.07	0.03	0.23
DI06	Summer *T*	27.05	0.29 ± 0.05	0.08	0.26
	Early summer *T*	26.41	0.28 ± 0.05	0.08	0.26
	Late summer *T*	16.33	0.23 ± 0.05	0.06	0.26
	Early winter *T*	7.92	0.17 ± 0.05	0.03	0.26
	Prev. fall *P*	5.55	0.15 ± 0.05	0.02	0.27
	Winter *T*	4.03	0.14 ± 0.06	0.02	0.26
DI07	Summer *T*	16.83	0.27 ± 0.06	0.08	0.23
	Early summer *T*	14.89	0.26 ± 0.06	0.07	0.23
	Late summer *T*	11.67	0.22 ± 0.06	0.06	0.23
	Late winter *T*	3.42	0.18 ± 0.08	0.03	0.23
	Winter *T*	2.15	0.15 ± 0.07	0.02	0.23
DI08	Summer *T*	21.85	0.38 ± 0.07	0.14	0.37
	Early summer *T*	19.5	0.36 ± 0.07	0.13	0.37
	Late summer *T*	15.41	0.32 ± 0.07	0.1	0.36
DI09	Late summer *T*	9.65	0.23 ± 0.07	0.07	0.27
	Summer *T*	5.27	0.20 ± 0.07	0.04	0.27
	Early summer *T*	2.89	0.17 ± 0.07	0.03	0.27
	Late summer *P*	2.13	−0.19 ± 0.09	0.02	0.27
DI10	Summer *T*	22.02	0.26 ± 0.05	0.08	0.24
	Late summer *T*	21.59	0.25 ± 0.05	0.08	0.24
	Early summer *T*	20.05	0.25 ± 0.05	0.07	0.25
	Prev. summer *T*	9.11	0.18 ± 0.05	0.04	0.24
	Winter *T*	6.94	0.18 ± 0.06	0.03	0.23
	Late winter *T*	5.02	0.16 ± 0.06	0.02	0.23
	Early winter *T*	3.55	0.14 ± 0.06	0.02	0.23
	Mid winter *T*	3.37	0.14 ± 0.06	0.02	0.23
	Early summer *P*	2.5	−0.13 ± 0.06	0.01	0.24
DI11	Summer *T*	9.93	0.24 ± 0.07	0.06	0.19
	Early summer *T*	9.76	0.23 ± 0.07	0.06	0.19
	Late summer *T*	3.99	0.17 ± 0.07	0.03	0.19
DI12	Late summer *T*	4.45	0.15 ± 0.06	0.03	0.23
	Summer *T*	4.32	0.16 ± 0.06	0.03	0.23
	Early summer *T*	4.16	0.16 ± 0.06	0.03	0.23
DI13	Summer *T*	3.06	0.18 ± 0.08	0.04	0.15
	Winter *T*	2.94	0.19 ± 0.09	0.04	0.14
	Mid winter *T*	2.46	0.18 ± 0.08	0.03	0.13
	Prev. fall *P*	2.32	0.16 ± 0.08	0.03	0.14
DI14	Late summer *T*	18.07	0.27 ± 0.06	0.09	0.23
	Summer *T*	14.76	0.26 ± 0.06	0.07	0.24
	Early summer *T*	10.18	0.22 ± 0.06	0.05	0.24
	Late winter *T*	8.79	0.24 ± 0.07	0.05	0.24
	Winter *T*	7.07	0.21 ± 0.07	0.04	0.24
	Mid winter *T*	3.97	0.17 ± 0.07	0.03	0.23
DI15	Summer *T*	13.68	0.28 ± 0.07	0.08	0.2
	Early summer *T*	11.03	0.26 ± 0.07	0.07	0.2
	Late summer *T*	7.45	0.21 ± 0.07	0.05	0.2
	Early winter *T*	3.5	0.18 ± 0.08	0.03	0.2
	Winter *T*	2.72	0.17 ± 0.08	0.03	0.19
DI16	Summer *T*	23.12	0.33 ± 0.06	0.12	0.37
	Early summer *T*	20.13	0.31 ± 0.06	0.10	0.37
	Late summer *T*	15.32	0.28 ± 0.06	0.08	0.36
DI17	Summer *T*	10.58	0.23 ± 0.06	0.06	0.28
	Early summer *T*	9.85	0.23 ± 0.06	0.06	0.28
	Late summer *T*	7.73	0.20 ± 0.06	0.05	0.28
	May *P*	3.74	0.17 ± 0.07	0.03	0.28
DI18	Summer *T*	13.82	0.27 ± 0.06	0.08	0.17
	Early summer *T*	11.82	0.26 ± 0.07	0.07	0.17
	Late summer *T*	10.34	0.23 ± 0.06	0.07	0.18
DI19	Summer *T*	23.83	0.30 ± 0.06	0.09	0.31
	Early summer *T*	21.04	0.28 ± 0.06	0.08	0.31
	Late summer *T*	15.2	0.24 ± 0.06	0.07	0.31
	May *T*	2.82	0.13 ± 0.06	0.02	0.31
	Winter *T*	2.82	0.14 ± 0.06	0.02	0.31
	Mid winter *T*	2.74	0.14 ± 0.06	0.02	0.31
DI20	Late summer *T*	6.14	0.20 ± 0.07	0.05	0.35
	Summer *T*	5.1	0.20 ± 0.07	0.04	0.35
	Winter *T*	4.11	0.21 ± 0.08	0.04	0.34
	Late winter *T*	3.93	0.21 ± 0.08	0.04	0.34
	Early summer *T*	3.25	0.17 ± 0.08	0.03	0.35
DI21	Late summer *T*	12.22	0.30 ± 0.08	0.10	0.28
	Summer *T*	11.2	0.30 ± 0.08	0.10	0.28
	Early summer *T*	7.67	0.26 ± 0.08	0.07	0.27
	Late winter *T*	4.99	0.26 ± 0.10	0.06	0.26
	Winter *T*	4.2	0.23 ± 0.09	0.05	0.25
	Prev. fall *P*	3.61	0.19 ± 0.08	0.05	0.27
	Mid winter *T*	3.54	0.21 ± 0.09	0.05	0.25
DI22	Late summer *T*	20.59	0.27 ± 0.05	0.08	0.27
	Summer *T*	16.32	0.25 ± 0.06	0.07	0.26
	Early summer *T*	13	0.23 ± 0.06	0.05	0.26
DI23	Summer *T*	18.63	0.36 ± 0.08	0.14	0.45
	Late summer *T*	16.94	0.34 ± 0.07	0.13	0.44
	Early summer *T*	15.46	0.34 ± 0.08	0.12	0.46
	Winter *T*	10.97	0.33 ± 0.09	0.10	0.44
	Early winter *T*	8.66	0.28 ± 0.08	0.08	0.44
	Mid winter *T*	7.16	0.28 ± 0.09	0.07	0.45
	Prev. summer *T*	4.51	0.21 ± 0.08	0.05	0.45
	Late winter *T*	3.95	0.24 ± 0.10	0.05	0.43
DI24	Late summer *T*	38.18	0.33 ± 0.05	0.13	0.21
	Summer *T*	25.41	0.30 ± 0.05	0.10	0.2
	Early summer *T*	15.78	0.25 ± 0.05	0.07	0.2
	Prev. summer *T*	4.93	0.16 ± 0.06	0.03	0.21
	Winter *T*	2.19	0.14 ± 0.07	0.02	0.19
	May *P*	2.16	0.13 ± 0.07	0.02	0.2
DI25	Late summer *T*	6.71	0.19 ± 0.06	0.04	0.25
	Winter *P*	6.3	−0.21 ± 0.07	0.04	0.26
	Summer *T*	5.51	0.19 ± 0.07	0.04	0.25
	Early winter *P*	5.15	−0.19 ± 0.07	0.04	0.26
	Early summer *T*	4.31	0.17 ± 0.07	0.03	0.25

Shrub	Model	∆AIC	Slope ± SE	mR^2^	cR^2^
*(b) 1893–1983*					
DI01	Early summer *T*	2.37	0.18 ± 0.09	0.03	0.27
DI02	Early summer *T*	6	0.24 ± 0.08	0.04	0.28
	Summer *T*	4.25	0.22 ± 0.09	0.04	0.28
DI03	–	–	–	–	–
DI04	Prev. summer *P*	3.79	0.28 ± 0.11	0.06	0.06
	Prev. summer *T*	2.48	−0.27 ± 0.13	0.05	0.06
DI05	Summer *T*	16.74	0.41 ± 0.08	0.11	0.11
	Late summer *T*	12.13	0.30 ± 0.07	0.09	0.12
	Early summer *T*	11.7	0.34 ± 0.08	0.08	0.11
DI06	Summer *T*	21.79	0.34 ± 0.07	0.08	0.19
	Early summer *T*	19.94	0.31 ± 0.06	0.08	0.19
	Prev. fall *P*	11.23	0.22 ± 0.06	0.05	0.21
	Late summer *T*	10.76	0.24 ± 0.06	0.05	0.19
	Winter *T*	6.14	0.19 ± 0.07	0.03	0.19
	Mid winter *T*	4.39	0.17 ± 0.07	0.02	0.19
	Late winter *T*	3.79	0.17 ± 0.07	0.02	0.18
DI07	Late winter *T*	4.73	0.28 ± 0.10	0.05	0.15
	Early summer *T*	2.75	0.20 ± 0.09	0.03	0.15
	Summer *T*	2.63	0.21 ± 0.10	0.03	0.14
DI08	Summer *T*	12.6	0.50 ± 0.12	0.13	0.20
	Early summer *T*	10.9	0.46 ± 0.12	0.12	0.21
	Late summer *T*	7	0.33 ± 0.10	0.09	0.18
	Mid winter *P*	2.04	−0.20 ± 0.10	0.04	0.22
DI09	Summer *P*	6.68	−0.39 ± 0.13	0.09	0.16

Summer *T*	4.13	0.32 ± 0.12	0.07	0.13

Late summer *P*	3.35	−0.29 ± 0.13	0.06	0.17

Late summer *T*	3.06	0.24 ± 0.10	0.06	0.14

Early summer *T*	2.88	0.28 ± 0.12	0.05	0.14

Early summer *P*	2.44	−0.27 ± 0.13	0.05	0.17
DI10	Early summer *T*	18.4	0.31 ± 0.07	0.07	0.12
	Summer *T*	15.32	0.30 ± 0.07	0.06	0.12
	Early summer *P*	9.92	−0.21 ± 0.06	0.04	0.13
	Late summer *T*	9.2	0.22 ± 0.06	0.04	0.12
	Summer *P*	5.36	−0.18 ± 0.06	0.03	0.11
	Late winter *T*	5.22	0.20 ± 0.07	0.03	0.11
	Winter *T*	3.77	0.17 ± 0.07	0.02	0.11
	Prev. summer *T*	3.16	0.17 ± 0.07	0.02	0.12
DI11	Summer *T*	7.66	0.33 ± 0.10	0.07	0.07
	Early summer *T*	6.14	0.30 ± 0.10	0.06	0.06
	Late summer *T*	3.95	0.21 ± 0.08	0.04	0.05
	Prev. summer *P*	2.16	0.22 ± 0.11	0.03	0.03
DI12	Summer *P*	5.55	−0.26 ± 0.09	0.05	0.17
	Early summer *P*	4.84	−0.23 ± 0.09	0.04	0.18
DI13	–	–	–	–	–
DI14	Summer *T*	11.89	0.33 ± 0.09	0.07	0.07
	Late summer *T*	9.98	0.26 ± 0.07	0.06	0.06
	Early summer *T*	9.83	0.29 ± 0.08	0.06	0.06
	Late winter *T*	8.87	0.29 ± 0.09	0.06	0.06
	Summer *P*	4.2	−0.23 ± 0.09	0.03	0.04
	Winter *T*	3.56	0.20 ± 0.08	0.03	0.03
	May *T*	2.9	0.21 ± 0.09	0.03	0.03
	Late summer *P*	2.7	−0.19 ± 0.09	0.03	0.03
DI15	–	–	–	–	–
DI16	Summer *T*	9.82	0.32 ± 0.09	0.07	0.20
	Early summer *T*	7.9	0.28 ± 0.09	0.06	0.21
	Late summer *T*	4.88	0.22 ± 0.08	0.04	0.20
	Prev. summer *P*	3.2	0.19 ± 0.08	0.03	0.19
	Mid winter *T*	2.64	0.20 ± 0.09	0.03	0.19
DI17	Early summer *T*	4.76	0.29 ± 0.11	0.06	0.33
	Summer *T*	4.52	0.30 ± 0.11	0.06	0.33
DI18	Summer *T*	3.35	0.33 ± 0.14	0.06	0.06
	Early summer *T*	2.67	0.30 ± 0.14	0.06	0.06
DI19	Summer *T*	13.16	0.31 ± 0.08	0.07	0.22
	Early summer *T*	12.76	0.30 ± 0.07	0.07	0.22
	May *T*	8.91	0.26 ± 0.08	0.05	0.22
	Late summer *T*	4.69	0.20 ± 0.08	0.03	0.22
	Winter *T*	2.62	0.17 ± 0.08	0.02	0.22
DI20	Late summer *T*	5.46	0.28 ± 0.10	0.07	0.28
	Winter *T*	2.88	0.28 ± 0.12	0.05	0.27
	Early winter *T*	2.23	0.23 ± 0.11	0.04	0.28
	Summer *T*	2.2	0.26 ± 0.12	0.04	0.28
DI21	Late summer *T*	4.53	0.37 ± 0.14	0.14	0.49

Summer *T*	3.28	0.39 ± 0.17	0.11	0.46

Winter *P*	2.98	−0.29 ± 0.13	0.10	0.45

Late winter *T*	2.77	0.43 ± 0.19	0.12	0.39

Mid winter *P*	2.17	−0.29 ± 0.14	0.10	0.50
DI22	Late summer *T*	11.89	0.25 ± 0.06	0.06	0.19
	Summer *T*	8.99	0.25 ± 0.07	0.05	0.17
	Early summer *T*	7.06	0.22 ± 0.07	0.04	0.16
	Early summer *P*	2.54	−0.13 ± 0.06	0.02	0.19
DI23	Summer *T*	14.81	0.52 ± 0.12	0.20	0.55
	Early summer *T*	13.73	0.51 ± 0.12	0.19	0.56
	Late summer *T*	7.57	0.37 ± 0.12	0.13	0.54
	Early winter *T*	5.07	0.33 ± 0.12	0.10	0.53
	Prev. summer *T*	4.08	0.32 ± 0.13	0.08	0.56
	Winter *T*	3.75	0.35 ± 0.14	0.08	0.55
	Mid winter *T*	2.64	0.32 ± 0.15	0.07	0.56
DI24	Late summer *T*	8.8	0.27 ± 0.08	0.06	0.06
	Early summer *P*	7.91	−0.26 ± 0.08	0.06	0.06
	Summer *P*	5.15	−0.25 ± 0.09	0.04	0.04
	Summer *T*	3.96	0.24 ± 0.10	0.04	0.04
DI25	Summer *P*	9.96	−0.34 ± 0.09	0.09	0.16
	Winter *P*	9.04	−0.28 ± 0.08	0.08	0.20
	Early summer *P*	8.25	−0.30 ± 0.09	0.07	0.16
	Early winter *P*	4.22	−0.19 ± 0.08	0.05	0.19
	Late winter *P*	4.12	−0.21 ± 0.09	0.04	0.21
	Summer *T*	3.35	0.25 ± 0.10	0.04	0.16
	Late summer *P*	2.74	−0.21 ± 0.10	0.04	0.17
	Winter *T*	2.7	0.25 ± 0.11	0.04	0.18
	Early summer *T*	2.38	0.22 ± 0.10	0.04	0.17
	Late summer *T*	2.35	0.19 ± 0.09	0.04	0.18

Shrub	Model	∆AIC	Slope ± SE	m*R* ^2^	c*R* ^2^
*(c) 1984–2013*					
DI01	–	–	–	–	–
DI02	Late summer *T*	0.23 ± 0.10	3.52	0.06	0.32
DI03	Prev. fall *T*	−0.17 ± 0.08	2.48	0.04	0.28
DI04	Prev. fall *T*	−0.16 ± 0.07	3.92	0.04	0.04
	Summer *P*	0.24 ± 0.10	3.16	0.03	0.03
DI05	–	–	–	–	–
DI06	Early winter *T*	0.23 ± 0.11	2.71	0.05	0.33
DI07	Summer *T*	0.22 ± 0.08	4.97	0.06	0.22
	Late summer *T*	0.20 ± 0.08	3.58	0.05	0.22
	Early summer *T*	0.20 ± 0.09	3.48	0.05	0.22
DI08	Summer *T*	0.25 ± 0.10	4.07	0.08	0.37
	Late summer *T*	0.24 ± 0.10	3.45	0.06	0.37
	Early summer *T*	0.23 ± 0.10	2.93	0.06	0.37
	Winter *T*	0.22 ± 0.11	2.14	0.05	0.37
DI09	Early summer *P*	0.30 ± 0.13	3.13	0.06	0.29
	Late summer *T*	0.20 ± 0.09	2.71	0.05	0.29
DI10	Late summer *T*	0.24 ± 0.09	5.55	0.07	0.31
	Winter *T*	0.21 ± 0.10	2.7	0.05	0.31
	Mid winter *T*	0.19 ± 0.09	2.01	0.04	0.31
DI11	–	–	–	–	–
DI12	Early summer *P*	0.25 ± 0.12	2.17	0.04	0.25
DI13	Early summer *P*	0.27 ± 0.13	2.41	0.05	0.16
	Prev. fall *T*	−0.17 ± 0.08	2.12	0.04	0.16
DI14	Early summer *P*	0.38 ± 0.13	6.26	0.09	0.31
	Late summer *T*	0.21 ± 0.09	3.06	0.06	0.31
	Summer *P*	0.28 ± 0.13	2.32	0.05	0.31
	Late winter *T*	0.22 ± 0.10	2.24	0.05	0.31
DI15	Summer *T*	0.23 ± 0.08	5.21	0.06	0.21
	Early summer *T*	0.21 ± 0.09	3.68	0.05	0.21
	Late winter *T*	0.20 ± 0.09	2.83	0.04	0.21
	Late summer *T*	0.18 ± 0.08	2.81	0.04	0.21
DI16	Summer *T*	0.30 ± 0.11	4.55	0.10	0.49
	Early summer *P*	0.40 ± 0.16	3.82	0.08	0.49
	Late summer *T*	0.28 ± 0.11	3.78	0.08	0.49
	Early summer *T*	0.28 ± 0.12	3.63	0.09	0.50
DI17	Late summer *T*	0.17 ± 0.07	3.66	0.04	0.15
	Summer *T*	0.16 ± 0.07	3.23	0.04	0.15
DI18	Summer *T*	0.22 ± 0.09	4.1	0.06	0.19
	Late summer *T*	0.19 ± 0.08	3.35	0.05	0.20
	Early summer *T*	0.20 ± 0.09	3.04	0.05	0.20
DI19	Early summer *P*	0.35 ± 0.13	5.41	0.07	0.32
	Late summer *T*	0.21 ± 0.09	3.24	0.06	0.32
DI20	Late winter *T*	0.24 ± 0.11	2.72	0.06	0.38
DI21	Late summer *T*	0.22 ± 0.09	3.94	0.06	0.19
	Summer *T*	0.23 ± 0.09	3.82	0.06	0.19
	Early summer *T*	0.20 ± 0.10	2.2	0.04	0.19
DI22	Late summer *T*	0.18 ± 0.09	2.34	0.04	0.25
DI23	Late summer *T*	0.30 ± 0.10	5.83	0.10	0.37
	Winter *T*	0.32 ± 0.11	5.75	0.10	0.37
	Late winter *T*	0.32 ± 0.12	5.42	0.10	0.37
	Mid winter *T*	0.26 ± 0.11	3.24	0.07	0.37
	Summer *T*	0.26 ± 0.11	3.24	0.07	0.37
DI24	Late summer *T*	0.34 ± 0.06	19.1	0.15	0.25
	Summer *T*	0.30 ± 0.07	11.43	0.10	0.25
	Early summer *T*	0.24 ± 0.08	6.09	0.07	0.25
	Late winter *P*	0.25 ± 0.09	4.75	0.06	0.25
	Winter *T*	0.21 ± 0.09	3.72	0.05	0.25
	Mid winter *T*	0.20 ± 0.09	3.32	0.04	0.25
	Late winter *T*	0.20 ± 0.09	2.69	0.04	0.25
DI25	–	–	–	–	–

Abbreviations: AIC, Akaike Information Criterion; cR^2^, conditional coefficient of determination (proportion of variance explained by fixed and random effects); mR^2^, marginal coefficient of determination (proportion of variance explained by fixed effect); *P*, precipitation sum; prev., previous; SE, standard error; *T*, mean temperature.

**TABLE A6:** Statistics of the selected climate‐growth models (∆AIC > 2) for individual shrubs calculated over the period 1964–2013.

Shrub	Model	∆AIC	Slope ± SE	mR^2^	cR^2^
DI01	Early summer *T*	6.91	0.22 ± 0.07	0.05	0.27
	Summer *T*	4.59	0.19 ± 0.07	0.04	0.27
	Prev. summer *P*	3.33	0.24 ± 0.10	0.03	0.28
DI02	Summer *T*	11.22	0.29 ± 0.08	0.09	0.33
	Early summer *T*	11.11	0.29 ± 0.08	0.09	0.33
	Late summer *T*	6.87	0.23 ± 0.08	0.06	0.33
	Early winter *T*	3.24	0.20 ± 0.09	0.04	0.33
	May *P*	2.35	0.22 ± 0.10	0.03	0.33
DI03	Early summer *T*	3.34	0.18 ± 0.08	0.03	0.30
	Winter *T*	3.06	0.19 ± 0.08	0.03	0.30
	Mid winter *T*	2.94	0.18 ± 0.08	0.03	0.30
	Early winter *T*	2.47	0.17 ± 0.08	0.03	0.30
	Summer *T*	2.36	0.16 ± 0.08	0.03	0.30
DI04	Prev. summer *P*	7.36	0.26 ± 0.08	0.04	0.04
	Prev. fall *T*	2.32	−0.13 ± 0.06	0.02	0.02
DI05	Summer *T*	12.27	0.28 ± 0.07	0.09	0.26
	Early summer *T*	12.19	0.28 ± 0.07	0.09	0.26
	Late summer *T*	7.5	0.23 ± 0.07	0.06	0.26
	Early winter *T*	2.06	0.17 ± 0.08	0.03	0.26
DI06	Summer *T*	11.99	0.28 ± 0.07	0.08	0.29
	Early summer *T*	11.98	0.28 ± 0.07	0.08	0.29
	Late summer *T*	10.08	0.26 ± 0.07	0.07	0.29
	Early winter *T*	4.22	0.20 ± 0.08	0.04	0.30
DI07	Summer *T*	16.3	0.30 ± 0.07	0.10	0.27
	Early summer *T*	13.81	0.29 ± 0.07	0.09	0.27
	Late summer *T*	10.84	0.25 ± 0.07	0.08	0.26
	Late winter *T*	2.19	0.18 ± 0.09	0.03	0.27
DI08	Summer *T*	19.15	0.38 ± 0.08	0.15	0.37
	Early summer *T*	17.12	0.36 ± 0.08	0.14	0.37
	Late summer *T*	14.89	0.34 ± 0.08	0.12	0.37
	Late winter *T*	3.11	0.24 ± 0.10	0.04	0.37
	Winter *T*	3.06	0.22 ± 0.10	0.04	0.37
	Prev. summer *P*	2.8	0.27 ± 0.12	0.04	0.37
DI09	Late summer *T*	11.39	0.27 ± 0.07	0.10	0.30
	Summer *T*	6.39	0.23 ± 0.08	0.06	0.30
	Early summer *T*	4.08	0.20 ± 0.08	0.04	0.30
DI10	Late summer *T*	15.28	0.28 ± 0.06	0.09	0.27
	Summer *T*	12.75	0.27 ± 0.07	0.08	0.28
	Early summer *T*	8.88	0.24 ± 0.07	0.06	0.28
	Winter *T*	6.91	0.23 ± 0.08	0.05	0.28
	Early winter *T*	4.85	0.20 ± 0.08	0.04	0.27
	Late winter *T*	4.13	0.21 ± 0.09	0.04	0.28
	Mid winter *T*	3.76	0.18 ± 0.08	0.04	0.27
	Prev. summer *T*	3.33	0.17 ± 0.07	0.03	0.27
DI11	Summer *T*	8.19	0.22 ± 0.07	0.06	0.19
	Early summer *T*	7.81	0.22 ± 0.07	0.05	0.19
	Late summer *T*	3.89	0.17 ± 0.07	0.03	0.19
DI12	Summer *T*	2.88	0.16 ± 0.07	0.03	0.23
	Late summer *T*	2.33	0.14 ± 0.07	0.03	0.23
	Early summer *T*	2.18	0.15 ± 0.07	0.02	0.23
DI13	Summer *T*	3.93	0.21 ± 0.09	0.05	0.16
	Mid winter *T*	2.54	0.19 ± 0.09	0.04	0.14
	Prev. fall *P*	2.45	0.18 ± 0.08	0.04	0.15
	Early summer *T*	2.32	0.18 ± 0.09	0.04	0.16
	Winter *T*	2.1	0.18 ± 0.09	0.03	0.14
DI14	Late summer *T*	12.88	0.28 ± 0.07	0.09	0.26
	Summer *T*	8.31	0.24 ± 0.07	0.07	0.26
	Late winter *T*	5.39	0.24 ± 0.09	0.05	0.26
	Early summer *T*	4.9	0.20 ± 0.08	0.05	0.26
	Winter *T*	4.46	0.21 ± 0.08	0.05	0.26
	Mid winter *T*	2.04	0.16 ± 0.08	0.03	0.26
DI15	Summer *T*	13.08	0.27 ± 0.07	0.08	0.20
	Early summer *T*	10.53	0.26 ± 0.07	0.07	0.20
	Late summer *T*	7.02	0.21 ± 0.07	0.05	0.19
	Winter *T*	2.64	0.17 ± 0.08	0.03	0.18
	Early winter *T*	2.19	0.16 ± 0.08	0.02	0.19
	May *P*	2.03	0.19 ± 0.09	0.03	0.19
DI16	Summer *T*	17.08	0.38 ± 0.08	0.15	0.43
	Late summer *T*	14.19	0.35 ± 0.08	0.13	0.43
	Early summer *T*	13.98	0.36 ± 0.08	0.13	0.44
	Winter *T*	2.05	0.20 ± 0.10	0.04	0.44
DI17	Summer *T*	10.18	0.26 ± 0.07	0.08	0.28
	Late summer *T*	9.64	0.25 ± 0.07	0.08	0.28
	Early summer *T*	9.17	0.25 ± 0.07	0.07	0.28
	Late winter *T*	2.01	0.19 ± 0.09	0.03	0.28
DI18	Summer *T*	14.86	0.30 ± 0.07	0.10	0.19
	Early summer *T*	13.52	0.29 ± 0.07	0.09	0.19
	Late summer *T*	9.21	0.24 ± 0.07	0.07	0.19
DI19	Summer *T*	13.14	0.31 ± 0.07	0.10	0.33
	Late summer *T*	10.08	0.27 ± 0.07	0.08	0.33
	Early summer *T*	10	0.28 ± 0.08	0.08	0.34
DI20	Late winter *T*	8.94	0.32 ± 0.09	0.08	0.36
	Winter *T*	6.94	0.27 ± 0.09	0.07	0.36
	Late summer *T*	6.31	0.23 ± 0.08	0.06	0.36
	Summer *T*	5.23	0.22 ± 0.08	0.05	0.36
	Mid winter *T*	3.98	0.22 ± 0.09	0.05	0.36
	Early summer *T*	3.15	0.19 ± 0.08	0.04	0.36
DI21	Late summer *T*	13.59	0.33 ± 0.08	0.13	0.30
	Summer *T*	13.21	0.33 ± 0.08	0.12	0.30
	Early summer *T*	9.56	0.30 ± 0.09	0.10	0.29
	Late winter *T*	5.44	0.28 ± 0.10	0.07	0.27
	Winter *T*	4.47	0.24 ± 0.09	0.06	0.26
	Mid winter *T*	3.31	0.21 ± 0.09	0.05	0.25
DI22	Summer *T*	14.97	0.31 ± 0.07	0.10	0.32
	Late summer *T*	14.09	0.30 ± 0.07	0.10	0.32
	Early summer *T*	13.74	0.31 ± 0.07	0.10	0.32
	Prev. summer *T*	3.38	0.18 ± 0.08	0.04	0.32
	Early winter *T*	2.42	0.18 ± 0.08	0.03	0.32
DI23	Late summer *T*	14.43	0.38 ± 0.09	0.15	0.44
	Late winter *T*	10.29	0.40 ± 0.11	0.12	0.44
	Winter *T*	10.1	0.36 ± 0.10	0.12	0.44
	Summer *T*	9.02	0.33 ± 0.10	0.11	0.44
	Early winter *T*	6.22	0.29 ± 0.10	0.08	0.44
	Early summer *T*	5.88	0.28 ± 0.10	0.08	0.44
	Mid winter *T*	4.23	0.26 ± 0.10	0.07	0.44
	Mid winter *P*	2.5	0.23 ± 0.11	0.05	0.44
DI24	Late summer *T*	32.5	0.35 ± 0.05	0.15	0.23
	Summer *T*	23.29	0.33 ± 0.06	0.12	0.23
	Early summer *T*	15.23	0.28 ± 0.06	0.09	0.23
	Winter *T*	5.06	0.20 ± 0.07	0.04	0.23
	Mid winter *T*	3	0.17 ± 0.07	0.03	0.22
	Early winter *T*	2.75	0.16 ± 0.07	0.03	0.23
	Late winter *T*	2.74	0.18 ± 0.08	0.03	0.23
	Late winter *P*	2.63	0.17 ± 0.08	0.03	0.23
	Prev. summer *T*	2.36	0.15 ± 0.07	0.02	0.23
	Prev. summer *P*	2.25	0.20 ± 0.10	0.02	0.23
DI25	Late summer *T*	5.36	0.21 ± 0.07	0.05	0.29
	Summer *T*	5.28	0.21 ± 0.08	0.05	0.29
	Early summer *T*	4.36	0.20 ± 0.08	0.05	0.29

Abbreviations: AIC, Akaike Information Criterion; cR^2^, conditional coefficient of determination (proportion of variance explained by the fixed and random effects); mR^2^, marginal coefficient of determination (proportion of variance explained by the fixed effect); *P*, precipitation sum; prev., previous; SE, standard error; *T*, mean temperature.
